# Persistence across Pleistocene ice ages in Mediterranean and extra-Mediterranean refugia: phylogeographic insights from the common wall lizard

**DOI:** 10.1186/1471-2148-13-147

**Published:** 2013-07-11

**Authors:** Daniele Salvi, D James Harris, Antigoni Kaliontzopoulou, Miguel A Carretero, Catarina Pinho

**Affiliations:** 1 CIBIO, Centro de Investigação em Biodiversidade e Recursos Genéticos, Universidade do Porto; Campus Agrário de Vairão, 4485-661, Vairão, Portugal

**Keywords:** *Podarcis muralis*, Phylogeography, Western Palaearctic, Glacial refugia, Mediterranean peninsulas, Genetic diversity, Temperate species

## Abstract

**Background:**

Pleistocene climatic oscillations have played a major role in structuring present-day biodiversity. The southern Mediterranean peninsulas have long been recognized as major glacial refugia, from where Northern Europe was post-glacially colonized. However, recent studies have unravelled numerous additional refugia also in northern regions. We investigated the phylogeographic pattern of the widespread Western Palaearctic lizard *Podarcis muralis*, using a range-wide multilocus approach, to evaluate whether it is concordant with a recent expansion from southern glacial refugia or alternatively from a combination of Mediterranean and northern refugia.

**Results:**

We analyzed DNA sequences of two mitochondrial (*cytb* and *nd4*) and three nuclear (*acm4*, *mc1r*, and *pdc*) gene fragments in individuals from 52 localities across the species range, using phylogenetic and phylogeographic methods. The complex phylogeographic pattern observed, with 23 reciprocally monophyletic allo- parapatric lineages having a Pleistocene divergence, suggests a scenario of long-term isolation in multiple ice-age refugia across the species distribution range. Multiple lineages were identified within the three Mediterranean peninsulas – Iberia, Italy and the Balkans - where the highest genetic diversity was observed. Such an unprecedented phylogeographic pattern - here called “refugia within *all* refugia” – compasses the classical scenario of multiple southern refugia. However, unlike the southern refugia model, various distinct lineages were also found in northern regions, suggesting that additional refugia in France, Northern Italy, Eastern Alps and Central Balkans allowed the long-term persistence of this species throughout Pleistocene glaciations.

**Conclusions:**

The phylogeography of *Podarcis muralis* provides a paradigm of temperate species survival in Mediterranean and extra-Mediterranean glacial refugia. Such refugia acted as independent biogeographic compartments for the long-term persistence of this species, for the differentiation of its genetic lineages, and for the short-distance post-glacial re-colonization of neighbouring areas. This finding echoes previous findings from recent phylogeographic studies on species from temperate ecoregions, thus suggesting the need for a reappraisal of the role of northern refugia for glacial persistence and post-glacial assembly of Holarctic biota.

## Background

Pleistocene climatic oscillations have played a major role in structuring current biodiversity patterns and shaping the distribution of species and their genetic diversity both at the regional and global scale [1,2]. In recent decades, phylogeographic studies have been used to assess the genetic consequences of Pleistocene ice ages on various organisms, highlighting the dynamic nature of species ranges and the role of microevolutionary processes in determining the extent and structure of intraspecific diversity [1,3,4].

In Europe, the genetic structure of temperate organisms has been mainly explained by cycles of contraction toward glacial refugia located in southern peninsulas and post-glacial recolonization of northern regions, tracking the expansion of suitable habitats [5,6] Accordingly, southern peninsular refugia are thought to have played a major role in the long-term maintenance of genetic diversity and differentiation, and relatively few patterns and routes of colonization have been described as paradigms for the post-glacial arrival of species in Northern Europe [6-8].

However, in recent years, multiple evidence from paleoclimatic, palaeontological and phylogeographic studies have identified the existence of glacial refugia in northern regions of Europe, or “northern refugia”, and have highlighted the prominent contribution of these refugia to the present-day genetic diversity of many organisms [9-12]. Mounting examples from plants, insects, mammals, amphibians, and reptiles show that in many temperate species the southern refugia model is insufficient to explain the observed genetic patterns, which would be better explained by a scenario of ice-age survival in a combination of Mediterranean and extra-Mediterranean refugia [13-17]. This changing view with respect to geographical location of refugia has also had direct implications for the inferred scenarios of recolonization for many widespread species from the Western Palaearctic. This is true even in emblematic species, such as the brown bear and the common beech, the phylogeographic patterns of which were previously upheld as paradigms of post-glacial recolonization routes (exclusively) from southern refugia [6,7]. In these species, the existence of additional extra-Mediterranean refugia has recently become evident through the analysis of fossil DNA and palaeobotanical data, respectively. These evidence suggests that range shifts and expansions in these species could have been much smaller than previously thought [18-21].

**Figure 1:**
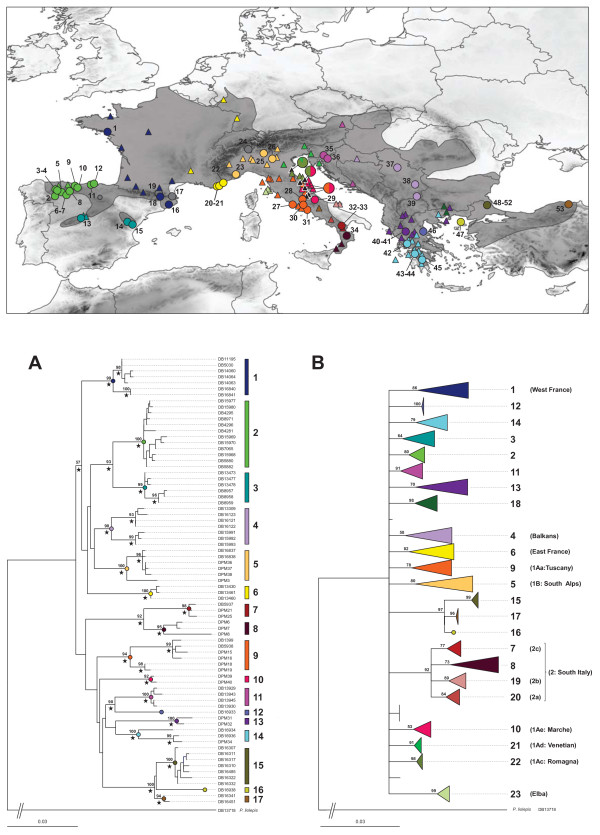
**Sampling localities**, **phylogenetic relationships**, **and distribution of mitochondrial haplotypes and haploclades.** Maximum Likelihood (ML) trees depicting the phylogenetic relationships among 75 combined mitochondrial sequences (*cytb* and *nd4*) of *Podarcis muralis* from 52 localities **(A)** and among 185 *cytb* sequences of *Podarcis muralis* obtained in this study (N=82) and from GenBank, (N=103) (see Additional file 1) **(B)**. ML bootstrap values over 1000 replicates are reported in correspondence to the nodes; black stars indicate BA posterior probabilities = 1.00; *Podarcis liolepis* was used as an outgroup. In the combined *cytb*/*nd4* ML tree specimen codes are reported (see Table 1 for details) and main mitochondrial clades numbered from 1 to 17; in the *cytb* ML tree tip nodes are collapsed and main mitochondrial clades are numbered from 1 to 23 according to the combined *cytb*/*nd4* ML tree and named according to previous studies. Samples used in this study and geographic distribution of mitochondrial clades are reported in the map: big circles show the 53 localities sampled in this study and triangles show the geographic origin for the *cytb* sequences obtained from GenBank, coloured according to mitochondrial clades defined by the ML trees (black triangles indicate admixed populations for which the frequency of haplotypes belonging to each clades is shown by the pie diagrams; grey circles indicate samples for which only nuclear sequences were available).

Detailed phylogeographic data from widespread Western Palaearctic species are particularly valuable for evaluating the plausibility of a scenario of ice-age survival in Mediterranean and extra-Mediterranean refugia. The common Wall lizard, *Podarcis muralis* (Laurenti, 1768) is a Western Palaearctic species which provides a suitable case-study for this purpose. *Podarcis muralis* is a locally abundant lacertid lizard found in a variety of environments across a wide altitudinal range (from sea level to over 2000 m of altitude). It exhibits considerable variation in colour patterns, biometry and pholidosis, which has led to the description of several subspecies [22,23]. The distribution of *P*. *muralis* is unusual relative to that of its congeners and, together with other characteristics, makes this species a useful model for evaluating the relative contribution of southern versus extra-Mediterranean refugia in shaping the current distribution of species and their genetic diversity. Firstly, it covers a wide range across the Western Palaearctic– from North-Eastern Anatolia and the Black Sea coast through all the Mediterranean peninsulas – Iberia, Italy, the Balkans– as well as across extra-Mediterranean regions of France, Germany, Slovenia, Austria and the Central Balkans (Figure 1). Secondly, it is abundant in both Mediterranean and continental climatic conditions and its distribution does not seem to be strongly limited by the presence of other lizards. Throughout the southern peninsulas *P*. *muralis* occurs in sympatry with more localized *Podarcis* species, such as members of the *P*. *hispanica* group in the Iberian Peninsula, *P*. *sicula* in the Italian Peninsula, and *P*. *erhardii* in the Balkans as well as with members of the genera *Lacerta*, *Iberolacerta*, and *Timon* across its whole range. Also, it is a highly successful invasive species in North-Western Europe where around 150 non-native *P*. *muralis* populations have been identified [24]. Thus, Mediterranean and extra-Mediterranean areas seem generally suitable for the survival and colonization of this species. Moreover, although little is presently known about the evolutionary history of *P*. *muralis*, either a scenario of recent colonization of its northern range from a southern primary range or a long-term history of the species in some of these northern areas can be invoked to explain the observed intraspecific variation. Harris and Arnold [25] hypothesized that *P*. *muralis* recently colonized most of its distribution from the Italian Peninsula, based on the observation that it exhibits the greatest morphological and allozyme diversity in Italy [26]. On the other hand, recent genetic assessment identified divergent genetic lineages - likely of Pleistocene origin - in Italy but also in France and the Balkans, disproving a colonization of these two latter areas from Italy [24,27,28]. Nevertheless, these studies have been limited to small parts of the range and analyzed only mitochondrial DNA, and particularly partial cytochrome *b* sequences, thus preventing a full appreciation of the origin of these lineages and of the overall evolutionary history and biogeography of the species.

In this study, we analysed DNA sequences data from 86 individuals from 52 localities across the species range, including extensive sampling in the Mediterranean peninsulas where native diversity is expected to be highest. We used two partial mitochondrial genes (*cytb* and *nd4*), and three partial nuclear genes (*acm4*, *mc1r*, and *pdc*), all of which have been successfully used for assessing variation within other species of lacertids (e.g. [29,30]). Furthermore, by comparing 103 published cytochrome *b* sequences from previous studies with our assessment, we placed these in a more robust phylogenetic framework, and further used them to confirm that all known diversity was included in this study. This comprehensive data set enables us to evaluate whether the patterns of genetic variation observed in this species reflect a recent expansion from a single or a few Pleistocene southern glacial refugia or, alternatively, from a combination of Mediterranean and extra-Mediterranean refugia. Under the first scenario we expect to identify endemic lineages in one or more southern refugia, to observe only southern-derived haplotypes in northern areas, and an overall pattern of ‘southern richness *vs* northern purity’ of genetic diversity [1,2,6]. Alternatively, if *P*. *muralis* also survived in northern glacial refugia, we predict an occurrence of lineages endemic to northern regions, with an ancient (Pleistocene) divergence from southern lineages, and an absence of a northern purity pattern of genetic diversity. The main goal of our range-wide multilocus approach is, therefore, to identify the biogeographic and evolutionary processes shaping the genetic diversity of *P*. *muralis* and to discuss, in the framework of European phylogeography, how this species came to have such an unusual biogeographic pattern.

## Methods

### Data collection

We sampled a total of 86 individuals from 52 localities spanning the range of *Podarcis muralis* with particular attention to the Iberian, Italian, and Balkan peninsulas. Three specimens of two Iberian *Podarcis*, *P*. *liolepis* (Boulenger, 1905) and *P*. *bocagei* (Seoane, 1884), and the Italian wall lizard *P*. *sicula* (Rafinesque-Schmaltz, 1810), were sampled and designated as outgroups. We used multiple outgroups because the phylogenetic position of *P*. *muralis* within the genus *Podarcis* is not well resolved, yet this species seems more closely related to Iberian *Podarcis* or *P*. *sicula*[31,32]. Additionally, partial *cytb* sequences for 103 individuals were obtained from GenBank and included in phylogenetic analyses. Specimens included in molecular analyses, together with collection locality details, are reported in Table 1 and Figure 1. Accession numbers for sequences retrieved from GenBank are reported in Additional file 1.

**Table 1:** **Geographical location and codes for the studied specimens of *Podarcis muralis *and the outgroup species *P***. ***liolepis***

**Specimen code**	**Locality code**	**Locality**		**Genbank accession numbers**				
			**Coordinates**	***cytb***	***nd4***	***mc1r***	***acm4***	***pdc***
DB16840	1	Vieille-Roche (France)	47.50 N -2.38 E	KF372191	KF372350	KF372113	KF372273	KF372036
DB16841	1	Vieille-Roche (France)	47.50 N -2.38 E	KF372192	KF372351	KF372114	KF372274	KF372037
DB4296	2	Palacios de Compludo (Spain)	42.46 N -6.47 E	KF372193	KF372352	KF372115	KF372275	KF372038
DB15980	3	Braña de Sosas (Spain)	43.00 N -6.31 E	KF372194	KF372353	KF372116	KF372276	KF372039
DB15977	4	Señora de Carrasconte (Spain)	42.94 N -6.23 E	KF372195	KF372354	KF372117	KF372277	KF372040
DB4281	5	La Omañuela (Spain)	42.78 N -5.98 E	KF372196	KF372355	KF372118	-	-
DB4295	6	León (Spain)	42.59 N -5.58 E	KF372197	KF372356	KF372119	KF372278	KF372041
DB15968	7	La Candamia (Spain)	42.60 N -5.55 E	KF372198	KF372357	KF372120	KF372279	KF372042
DB15969	8	Valdehuesa (Spain)	42.94 N -5.32 E	KF372199	KF372358	KF372121	KF372280	KF372043
DB15970	8	Valdehuesa (Spain)	42.94 N -5.32 E	KF372200	KF372359	KF372122	KF372281	KF372044
DB8970	9	Tanes (Spain)	43.21 N -5.40 E	KF372201	-	KF372123	KF372282	KF372045
DB8971	9	Tanes (Spain)	43.21 N -5.40 E	KF372202	KF372360	KF372124	KF372283	KF372046
DB7092	10	Turieno (Spain)	43.16 N -4.66 E	-	KF372361	KF372125	KF372284	KF372047
DB5880	11	Matienzo (Spain)	43.33 N -3.59 E	KF372203	KF372362	KF372126	KF372285	KF372048
DB5882	11	Matienzo (Spain)	43.33 N -3.59 E	KF372204	KF372363	KF372127	KF372286	KF372049
DB7065	12	Oriñon (Spain)	43.40 N -3.33 E	KF372205	KF372364	KF372128	KF372287	KF372050
DB8957	13	Guadarrama (Spain)	40.71 N -4.14 E	KF372206	KF372365	KF372129	KF372288	KF372051
DB8958	13	Guadarrama (Spain)	40.71 N -4.14 E	KF372207	KF372366	KF372130	KF372289	KF372052
DB8959	13	Guadarrama (Spain)	40.71 N -4.14 E	KF372208	KF372367	-	-	-
DB13472	14	Gúdar (Spain)	40.37 N -0.67 E	-	-	KF372131	-	KF372053
DB13473	14	Gúdar (Spain)	40.37 N -0.67 E	KF372209	KF372368	KF372132	KF372290	KF372054
DB13477	15	Penyagolosa (Spain)	40.23 N -0.35 E	KF372210	KF372369	KF372133	KF372291	KF372055
DB13478	15	Penyagolosa (Spain)	40.23 N -0.35 E	KF372211	KF372370	KF372134	KF372292	KF372056
DB14060	16	Montseny (Spain)	41.77 N 2.44 E	KF372212	KF372371	KF372135	KF372293	KF372057
DB14063	16	Montseny (Spain)	41.77 N 2.44 E	KF372213	KF372372	KF372136	KF372294	KF372058
DB14064	16	Montseny (Spain)	41.77 N 2.44 E	KF372214	KF372373	KF372137	KF372295	KF372059
DB1759	17	Les Salines (Spain)	42.42 N 2.75 E	KF372215	-	-	-	-
DB5030	18	Meranges (Spain)	42.43 N 1.78 E	KF372216	KF372374	KF372138	KF372296	KF372060
DB11195	18	Meranges (Spain)	42.43 N 1.78 E	KF372217	KF372375	KF372139	KF372297	-
DB13718*	19	Luzenac (France)	42.75 N 1.75 E	KF372218	KF372376	-	-	-
DB13461	20	Massif des Maures (France)	43.24 N 6.38 E	KF372219	KF372377	KF372140	KF372298	KF372061
DB13460	21	Valle de Gilly (France)	43.28 N 6.46 E	KF372220	KF372378	KF372141	KF372299	KF372062
DB13430	22	Massif des Maures (France)	43.38 N 6.62 E	KF372221	KF372379	KF372142	KF372300	KF372063
DPM1	23	Viozene (Italy)	44.14 N 7.78 E	KF372222	-	KF372143	KF372301	KF372064
DPM3	23	Viozene (Italy)	44.14 N 7.78 E	KF372223	KF372380	KF372144	KF372302	KF372065
DB15936	24	Monte Verita (Switzerland)	46.16 N 8.72 E	KF372224	-	-	-	-
DB16837	25	Bianzano (Italy)	45.77 N 9.92 E	KF372225	KF372381	KF372145	KF372303	KF372066
DB16838	25	Bianzano (Italy)	45.77 N 9.92 E	KF372226	KF372382	KF372146	KF372304	-
DPM36	26	Peschiera del Garda (Italy)	45.44 N 10.67 E	KF372227	KF372383	KF372147	KF372305	KF372067
DPM37	26	Peschiera del Garda (Italy)	45.44 N 10.67 E	KF372228	KF372384	-	KF372306	KF372068
DPM38	26	Peschiera del Garda (Italy)	45.44 N 10.67 E	KF372229	KF372385	KF372148	KF372307	KF372069
DB1399	27	Ostia Antica (Italy)	41.75 N 12.31 E	KF372230	KF372386	KF372149	KF372308	KF372070
DB5938	28	Paganico (Italy)	42.19 N 13.00 E	KF372231	KF372387	KF372150	KF372309	KF372071
DPM39	29	Majelletta (Italy)	42.16 N 14.13 E	KF372232	KF372388	KF372151	KF372310	KF372072
DPM40	29	Majelletta (Italy)	42.16 N 14.13 E	KF372233	KF372389	KF372152	KF372311	KF372073
DPM41	29	Majelletta (Italy)	42.16 N 14.13 E	KF372234	-	-	-	KF372074
DPM15	30	Bassiano (Italy)	41.55 N 13.05 E	KF372235	KF372390	KF372153	KF372312	KF372075
DPM16	30	Bassiano (Italy)	41.55 N 13.05 E	KF372236	KF372391	KF372154	KF372313	KF372076
DPM18	31	Fondi (Italy)	41.37 N 13.33 E	KF372237	KF372392	KF372155	KF372314	KF372077
DPM19	31	Fondi (Italy)	41.37 N 13.33 E	KF372238	KF372393	KF372156	KF372315	KF372078
DPM25	32	Pollino National Park (Italy)	39.93 N 16.17 E	KF372239	KF372394	KF372157	KF372316	KF372079
DB5937	33	Pollino National Park (Italy)	39.90 N 16.19 E	KF372240	KF372395	KF372158	KF372317	KF372080
DPM21	33	Pollino National Park (Italy)	39.90 N 16.19 E	KF372241	KF372396	KF372159	KF372318	KF372081
DPM7	34	Fago del Soldato (Italy)	39.35 N 16.55 E	KF372242	KF372397	KF372160	KF372319	KF372082
DPM8	34	Fago del Soldato (Italy)	39.35 N 16.55 E	KF372243	KF372398	KF372161	KF372320	KF372083
DPM6	34	Fago del Soldato (Italy)	39.35 N 16.55 E	KF372244	KF372399	KF372162	KF372321	KF372084
DB13929	35	Ribnica (Slovenia)	45.75 N 14.77 E	KF372245	KF372400	KF372163	KF372322	KF372085
DB13930	35	Ribnica (Slovenia)	45.75 N 14.77 E	KF372246	KF372401	KF372164	KF372323	KF372086
DB13943	36	Donja Lamana Draga (Slovenia)	45.51 N 14.96 E	KF372247	KF372402	KF372165	KF372324	KF372087
DB13945	36	Donja Lamana Draga (Slovenia)	45.51 N 14.96 E	KF372248	KF372403	KF372166	KF372325	KF372088
DB15991	37	Zuce (Serbia)	44.68 N 20.55 E	KF372249	KF372404	KF372167	KF372326	KF372089
DB15992	37	Zuce (Serbia)	44.68 N 20.55 E	KF372250	KF372405	KF372168	KF372327	KF372090
DB15993	37	Zuce (Serbia)	44.68 N 20.55 E	KF372251	KF372406	KF372169	KF372328	KF372091
DB13309	38	Cиheвo (Serbia)	43.34 N 22.08 E	KF372252	KF372407	KF372170	KF372329	KF372092
DB16121	39	Road to Crnovska River (Serbia)	42.38 N 22.05 E	KF372253	KF372408	KF372171	KF372330	KF372093
DB16122	39	Road to Crnovska River (Serbia)	42.38 N 22.05 E	KF372254	KF372409	KF372172	KF372331	KF372094
DB16123	39	Road to Crnovska River (Serbia)	42.38 N 22.05 E	KF372255	KF372410	KF372173	KF372332	KF372095
DPM31	40	Metsovo (Greeece)	39.71 N 21.19 E	KF372256	KF372411	KF372174	KF372333	KF372096
DPM32	41	Metsovo (Greeece)	39.71 N 21.21 E	KF372257	KF372412	KF372175	KF372334	KF372097
DB16934	42	Velouxi (Greece)	38.85 N 21.38 E	KF372258	KF372413	-	KF372335	KF372098
DPM34	43	Platanitsa, Achaia (Greece)	37.97 N 21.88 E	KF372259	KF372414	KF372176	KF372336	KF372099
DPM35	44	Platanitsa, Achaia (Greece)	37.96 N 21.91 E	KF372260	-	KF372177	KF372337	KF372100
DB16936	45	Mainalo, Pelloponnisos (Greece)	37.39 N 22.46 E	KF372261	KF372415	-	-	-
DB16933	46	Kisavos mt (Greece)	39.63 N 22.64 E	KF372262	KF372416	-	-	-
DB16938	47	Samothraki isl. (Greece)	40.45 N 25.59 E	KF372263	KF372417	KF372178	KF372338	-
DB16310	48	Kapakli (Turkey)	41.89 N 27.35 E	KF372264	KF372418	KF372179	KF372339	KF372101
DB16307	49	Kapakli (Turkey)	41.91 N 27.36 E	KF372265	KF372419	KF372180	KF372340	KF372102
DB16311	50	Dereköy (Turkey)	41.96 N 27.39 E	KF372266	KF372420	KF372181	KF372341	KF372103
DB16317	50	Dereköy (Turkey)	41.96 N 27.39 E	KF372267	KF372421	KF372182	KF372342	KF372104
DB16482	50	Dereköy (Turkey)	41.96 N 27.39 E	-	KF372422	KF372183	KF372343	KF372105
DB16322	51	Dereköy (Turkey)	41.97 N 27.42 E	KF372268	KF372423	KF372184	KF372344	KF372106
DB16332	51	Dereköy (Turkey)	41.97 N 27.42 E	KF372269	KF372424	KF372185	KF372345	KF372107
DB16485	51	Dereköy (Turkey)	41.97 N 27.42 E	KF372270	KF372425	KF372186	KF372346	KF372108
DB16318	52	Dereköy (Turkey)	41.96 N 27.45 E	-	KF372426	KF372187	KF372347	KF372109
DB16341	53	Pınarözü (Turkey)	41.77 N 34.04 E	KF372271	KF372427	KF372188	KF372348	KF372110
DB16340	53	Pınarözü (Turkey)	41.77 N 34.04 E	-	KF372428	KF372189	KF372349	KF372111
DB16451	53	Pınarözü (Turkey)	41.77 N 34.04 E	KF372272	KF372429	KF372190	-	KF372112

Specimen and code locality along with geographic coordinates are reported. Genbank Accession Numbers for each gene are provided. *outgroup species: *P. liolepis.*

Total genomic DNA was extracted from alcohol-preserved tail muscle collected from live specimens following standard high-salt protocols [33]. Portions of five genes were amplified by polymerase chain reaction: two mitochondrial genes, cytochrome *b* (*cytb*) and NADH dehydrogenase subunit 4 with flanking tRNAs (*nd4*), and three nuclear genes, Melanocortin receptor 1 (*mc1r*), acetylcholinergic receptor M4 (*acm4*), and Phosducin (*pdc*). The primers used for amplification and respective references are reported in Additional file 2. For *pdc* we used primers newly developed for this study. Amplifications were carried out in 25 μL volumes, containing 1X PCR buffer (50 mm Tris–HCl, 50 mm NaCl, pH 8.5); 3 mM MgCl_2_; 0.6 mM each dNTP, 2U of GoTaq DNA polymerase (Promega), 0.4 μM each primer and approximately 50 ng of genomic DNA. Amplification conditions consisted of a preliminary denaturation step at 92°C for 3 minutes, followed by 16 touchdown cycles with 30 seconds at 92°C, annealing temperature decreasing 0.5°C per cycle from 60°C to 52°C (30 seconds) and extension for 1 minute at 72°C. 20 more cycles similar to these but with annealing temperatures stable at 52°C followed. A final extension was carried out at 72°C for 15 minutes.

### Data analysis

Electropherograms were checked and consensus sequences were aligned in GENEIOUS 6.0 (http://www.geneious.com) using the Geneious Alignment algorithm. We discarded the hypothesis of pseudogenes occurring in our mitochondrial sequence dataset by confirming: (i) the absence of stop codons in protein-coding fragments, (ii) the consistency of the base composition pattern with a mitochondrial origin (less than 5% of guanines in the third position, see [34]), and (iii) the overall similarity with the reference mitochondrial genome of *P*. *muralis* (Genbank accession number FJ460597). Haplotype phase of nuclear genes was determined using PHASE version 2.1.1. [35]. Estimations were carried out under a model with recombination (−MR0 option), with the initial 1000 iterations discarded as burn-in, 1 as thinning interval and 1000 post-burnin iterations. Three independent runs were conducted for each gene. After monitoring the goodness of fit for each run according to the program’s manual, we accepted haplotype reconstructions which both i) yielded the same result in each of the three runs, and ii) had associated phase posterior probabilities of at least 0.75 in the average of the three runs. For each nuclear gene, the possible occurrence of recombination events was assessed using the Pairwise Homoplasy Index (PHI) test [36] implemented in SPLITSTREE 4.11 [37].

In order to assess patterns of genetic diversity and evaluate whether southern peninsulas harboured most of the diversity of *P*. *muralis*, we estimated the overall genetic diversity of the species and its distribution across populations and regions. For each gene we computed the following summary statistics of genetic diversity: number of segregating sites *S*, nucleotide diversity *π*, number of haplotypes *H*, and haplotype diversity *Hd*, both overall and for specific groups or clades defined within the species (see Results and Discussion) with DNASP 5 [38]. Comparisons of diversity measures can be greatly affected by different sample sizes among groups. In order to account for this, we followed a resampling approach: given the lowest sample size (except 0 or 1) amongst the various groups being compared (s), for each group with sample size > s we resampled 100 sets of s sequences, calculated diversity measures for each of these 100 sets and took the average for each diversity measure. This procedure allows a straightforward comparison of diversity measures derived from groups or clades with distinct sample sizes. Resampling was performed with the aid of a series of scripts written in Python 2.7.1 (available from the authors upon request) and taking advantage of DNASP “batch mode” calculations. Average uncorrected genetic distances between mitochondrial clades were assessed using MEGA 5 [39]. Furtehrmore, to assess genetic differentiation between clades we calculated *F*_ST_ (based on *p*-distance) and its significance by performing 1000 permutations in ARLEQUIN 3.5.1.2 [40].

Phylogenetic relationships among mitochondrial haplotypes were inferred using Maximum Likelihood (ML) and Bayesian (BA) methods. We tested three outgroups, *P*. *liolepis*, *P*. *bocagei*, and *P*. *sicula* (both combined and separately) through preliminary ML and BA tree searches. The outgroup effect on the ML and BA topologies was limited and differences between trees calculated using different outgroups were limited to unsupported nodes (not shown). Thus, we present and discuss phylogenetic analyses using *P*. *liolepis* as an outgroup (Genbank Accession numbers for *P*. *bocagei cytb*/*nd4*: DQ081139/DQ081153; for *P*. *sicula cytb/nd4*: KF372034/KF372035; for *P*. *liolepis* see Table 1). The ML search and the model selection were carried out in TREEFINDER version October 2008 [41]. We selected the best partitioned model of nucleotide substitution under the corrected Akaike’s Information Criterion (AICc) by comparing the AICc scores of seven different partition schemes (under a fixed ML topology): (i) by gene fragments (*cytb*/*nd4*+tRNAs), (ii) by genes (*cytb*/*nd4*/tRNAs); (iii) by coding regions (*cytb*+*nd4*/tRNAs), (iv-v) by codon position alone (two schemes:1st/2nd/3rd and 1st+2nd/3rd), and (vi-vii) by codon position, in combination with gene partitions (for a total of seven or five partitions, depending on the codon partitioning scheme). The optimal model had seven partitions, three codon partitions for each coding gene and one partition for tRNAs (*cytb*: HKY for the 1st, the 2nd, and the 3rd codon positions; *nd4*: TN+ G for the the 1st, and HKY+G for the 2nd and the 3rd codon positions; tRNAs: HKY+G, each model with frequency and rate heterogeneity parameters optimisation). We performed a global tree search using 100 random start trees generated through equidistant random walks of random nearest-neighbour-interchanges (NNI) starting from the centre tree obtained by a simple ML search. Support for the nodes (BP) was evaluated with 1000 bootstrap replicates. Additionally, in order to evaluate the total mitochondrial variation known for this species, we carried out a ML analysis following the same procedure as above (the best model in this case was the HKY with a single partition), using a dataset including both the *cytb* sequences generated in this study (N=82) and those available in GenBank for native populations of *P*. *muralis* (N=103). This dataset provides even greater coverage of the whole species range (see Figure 1). The obtained phylogenetic trees were visualised and edited using FigTree v.1.3.1 (available at http://tree.bio.ed.ac.uk/software/figtree/). The geographic pattern of haplogroup distribution and the relationships between haplotypes help to disentangle the two alternative scenarios predicted by the southern refugia model or by a model of Mediterranean and extra-Mediterranean refugia. For this purpose, we mapped the distribution of haplogroups identified by the phylogenetic trees across our range-wide sampling.

Bayesian analyses were carried out using the BEAST v.1.7.4 package [42] for two purposes: i) to estimate a phylogeny of the combined mitochondrial DNA dataset based on Bayesian inference; ii) to obtain estimates of the mtDNA time to the most recent common ancestor (TMRCA) of *P*. *muralis* and groups detected within the species. The choice of an appropriate tree prior for this dataset is somewhat problematic: on one hand, the bulk of our data is intraspecific, hence a “speciation” prior such as the Yule process may not be adequate. On the other hand, because the presence of an outgroup is mandatory and due to the high levels of geographic substructure detected in our focal species (see Results), our data are not totally amenable to analyses under a basic coalescent process. Therefore, we performed these analyses under both a Yule process and a coalescent process with constant size, to evaluate the effect of prior selection on our estimates of phylogeny and TMRCAs. To avoid overparametrization, we implemented a model with a simpler partition scheme, defined by gene fragments (two-partitions), and selected under AICc in TREEFINDER. The best evolutionary model for our dataset assumed the HKY+G and the TIM2+G+I substitution models for the *cytb* and *nd4* fragments, respectively. The TIM2 model was manually specified within the BEAUti input file [42]. To infer mtDNA TMRCAs, we assumed a relaxed molecular clock for both *cytb* and *nd4*, applying an uncorrelated lognormal model [43]. We used the available rates proposed for *Podarcis* for the longer fragment, *nd4*, while providing a large uninformative prior for the *cytb* substitution rate (parameter ucld.mean). In the case of *nd4* we assumed that differences accumulate at a mean rate of 2.26% per million years, obtained as the average between divergence rates proposed for *Podarcis* for this fragment (ranging from 1.74% to 2.78% per million years; [44]). In order to incorporate this uncertainty into TMRCA estimates, we defined a normal prior distribution on the mutation rate with mean 1.13%/MY and with 95% of the probability distribution encompassed between the two aforementioned limits (0.87 and 1.39%, respectively). For each tree prior, BEAST was run twice, with 100 million iterations per run, sampling every 10000 steps. TRACER v. 1.5 (available at http://beast.bio.ed.ac.uk/Tracer) was used to visualize the results and assess if the effective sample size of estimated parameters was satisfactory. We discarded the initial 10% of sampled trees as burn-in. Upon confirmation of convergence, the two outputs for each tree prior were combined and subsequent data were retrieved from the combined data set.

For the following analyses, we considered as clades groups of haplotypes which are reciprocally monophyletic at the mitochondrial loci with the ML and BA tree reconstructions; displaying geographical contiguity; and divergent by more than 1% with its closer relative in line with previous studies ([24,27,28]; see also [45]). While we acknowledge that such criteria could be considered arbitrary, to further test that these groups correspond to evolutionary significant lineages, ESU *sensu*[46], we confirmed they show significant divergence of allele frequencies at nuclear loci as assessed by *F*_ST_ statistics (see above).

To assess how genetic variance at the combined *cytb*/*nd4* sequences was hierarchically distributed among and within mitochondrial clades, we performed a spatial analysis of molecular variance (SAMOVA) with SAMOVA 1.0 [47]. We pooled samples from neighbouring localities in accordance with the mitochondrial clades previously identified. Geographic coordinates for each clade were calculated as the geographic centroid of member localities. Clades represented by less than two individuals were not included in the analysis. SAMOVA was run for 10,000 iterations from each of 100 random initial conditions, and testing the predefined number of groups (*K*) from 2 to 14.

Phylogenetic relationships among nuclear haplotypes were inferred by median joining networks [48] using the software NETWORK 4.6.1.0 (available at http://www.fluxus-engineering.com/sharenet.htm).

Finally, we specifically tested the hypothesis of a recent expansion of *P*. *muralis* from southern glacial refugia against the alternative scenario of Pleistocene survival of the species in both southern and northern refugia. The first hypothesis implies that northern localities belong to the same genetic pool as the southern region from which they originated. As such, sequence divergence between northern and southern localities will be, in most cases, significantly lower than the divergence observed between clades and possibly similar to intraclade divergence. By contrast, the latter hypothesis implies that northern regions have hosted surviving populations which have evolved independently from southern ones. Thus, we expect the mean sequence divergence between northern and southern localities to be similar to mean divergence among clades. This rationale provides a statistical framework to explicitly compare the two competing hypotheses. A requisite of this test is that mean between-clade sequence divergence is significantly higher than within-clade divergence. Therefore, as a first step, we performed a permutation test to evaluate if between-clade *p*-distances (*D*_*bc*_) were significantly higher than within-clade distances (*D*_*wc*_). For this purpose, we randomly shuffled individuals among clades 1000 times in order to obtain the empirical distribution of the difference *D*_*bc*_ – *D*_*wc*_. The corresponding *p*-value was then obtained as the percentage of times for which the observed *D*_*bc*_ – *D*_*wc*_ was smaller than random. This test was performed for the combined mitochondrial DNA data set, as well as for the three nuclear loci in separate, always using the units defined on the basis of the mtDNA phylogeny. In continuation, for each northern locality and for each locus, we evaluated if the sequence divergence from southern localities fell within the “between-clade” or the “within-clade” level of differentiation. We classified localities as “northern” or “southern” according to biogeographic barriers separating central Europe from major glacial refugia in southern peninsulas as defined by previous literature [1,4,5,7,15]: localities north or east to the Pyrenees (locality 1), north to the Appennine chain (localities 25–26), west or east to the Alps (localities 20–23 and 35–36, respectively), and in the central Balkans (localities 37–39) were therefore considered as “northern”. For each northern locality we performed two different tests: first, we examined its mean *p*-distance from all southern localities (localities 2–16, 18, 27–34, and 40–46). Second, to account for the possibility that northern localities may have been preferably colonized from adjacent refugia, we also examined the mean *p*-distance of each northern locality from contiguous southern regions only (locality 1 vs localities 2–16, and 18; localities 20–26 vs localities 27–34; localities 37–39 vs. localities 40–46; localities 35–36 were compared either with localities 27–34 or with localities 40–46, given their intermediate geographic location). These comparisons were performed according to the following permutation procedure: individuals were shuffled among localities at random across 1000 permutation cycles. In each permutation, we calculated the mean *p*-distance between each northern locality and the set of southern localities being compared (*D*_*i*_, where *i* is the code of the northern locality), the mean between-clade distance (*D*_*bc*_) and the mean within-clade distance (*D*_*wc*_). For each northern locality *i*, we then assessed whether the difference between *D*_*bc*_ and *D*_*i*_ (*D*_*bc*_ – *D*_*i*_) was equal or higher than the distance we observed in the real data. The same was done for the difference between *D*_*i*_ and *D*_*wc*_ (*D*_*i*_ - *D*_*wc*_). The proportion of permutations verifying each of these conditions was used as a *p*-value to assess if the observed differences were significant or could be due to chance alone.

## Results

We obtained 82 *cytb* mitochondrial sequences of 411 base pairs (bp), 79 *nd4* mitochondrial sequences of 873 bp (75 individuals sequenced for both fragments), 156 sequences for the nuclear gene fragment *mc1r*, and 154 for *acm4* and *pdc* with 688bp, 423 bp, and 342 bp, respectively. GenBank accession numbers for the sequences generated in this study are reported in Table 1. Multiple sequence alignment did not require gap positions in any of the studied genes. The phi test did not find statistically significant evidence for recombination within nuclear gene fragments (*p* > 0.05). Sample size and localities, and clade assignment are reported in Table 2.

**Table 2:** **Summary statistics of genetic diversity for each gene**, **clade**, **and region**

		***nd4***						***cytb***						***mc1r***						***acm4***						***pdc***					
**Clades**	**Localities**	**N**	**ns**	***S***	***h***	***Hd***	π (*10^**3**^**)**	**N**	**ns**	***S***	***h***	***Hd***	π (*10^**3**^**)**	**N**	**ns**	***S***	***h***	***Hd***	π (*10^**3**^**)**	**N**	**ns**	***S***	***h***	***Hd***	π (*10^**3**^**)**	**N**	**ns**	***S***	***h***	***Hd***	π (*10^**3**^**)**
**1**	1, 16, 18	7	873	8	5	0.91	4.67	8	411	5	3	0.61	5.20	14	683	3	3	0.28	0.74	14	423	1	2	0.26	0.82	12	342	2	2	0.30	2.45
			754	3.55	1.93	0.93	4.71		391	2.07	1.63	0.63	5.48		576	0.41	1.25	0.25	0.71		323	0.3	1.3	0.30	0.93		248	1.24	1.62	0.33	2.69
**2**	3-9, 11, 12	13	873	9	13	1.00	3.98	13	411	2	3	0.30	1.15	28	683	4	3	0.14	0.50	26	423	0	1	0.00	0.00	26	342	3	3	0.66	5.49
			754	3.05	2	1.00	4.05		391	0.47	1.33	0.33	1.24		576	0.54	1.24	0.24	0.94		323	0	1	0.00	0.00		248	2.41	2.41	0.68	5.56
**3**	13- 15	6	873	5	5	0.93	3.36	6	411	4	4	0.87	5.47	12	683	1	2	0.17	0.29	10	423	1	2	0.20	0.62	12	342	2	2	0.49	3.93
			754	2.41	1.92	0.92	3.20		391	2.09	1.9	0.90	5.53		576	0.15	1.15	0.15	0.26		323	0.2	1.2	0.20	0.62		248	1.66	1.83	0.47	3.82
**4**	37-39	7	873	7	6	0.95	4.93	7	411	7	4	0.81	9.07	14	683	3	3	0.39	1.16	14	423	2	3	0.56	2.35	14	342	4	3	0.48	6.41
			754	3.49	1.92	0.92	4.63		391	2.94	1.75	0.75	7.78		576	0.7	1.42	0.42	1.22		323	0.81	1.6	0.60	2.51		248	2.72	1.94	0.45	6.01
**5**	23, 25, 26	6	873	10	6	1	5.31	8	411	8	3	0.61	10.14	12	683	4	4	0.64	1.71	14	423	3	4	0.75	3.61	12	342	3	4	0.77	4.77
			754	3.81	2	1	5.05		391	3.70	1.53	0.53	9.46		576	0.96	1.61	0.61	1.67		323	1.29	1.77	0.77	3.99		248	2.18	2.79	0.77	4.89
**6**	20-22	3	873	1	2	0.67	0.88	3	411	2	3	1.00	3.53	6	683	0	1	0.00	0.00	6	423	1	2	0.60	1.86	6	342	0	1	0.00	0.00
			754	0.65	1.65	0.65	0.86		391	1.3	2	1.00	3.44		576	0	1	0.00	0.00		323	0.51	1.51	0.51	1.58		248	0	1	0.00	0.00
**7**	32, 33	3	873	3	3	1.00	3.10	3	411	1	2	0.67	1.76	6	683	1	2	0.33	0.58	6	423	3	5	0.93	4.33	6	342	3	3	0.60	4.05
			754	2.29	2	1.00	3.04		391	0.62	1.62	0.62	1.64		576	0.29	1.29	0.29	0.50		323	1.44	1.97	0.97	4.46		248	1.92	2.29	0.58	3.89
**8**	34	3	873	11	3	1.00	9.73	3	411	10	2	0.67	17.64	6	683	1	2	0.33	0.58	6	423	2	3	0.80	3.30	6	342	1	2	0.33	1.35
			754	7.54	2	1.00	10.00		391	7.5	1.75	0.75	19.84		576	0.29	1.29	0.29	0.50		323	1.05	1.78	0.78	3.25		248	0.62	1.62	0.31	1.25
**9**	27, 28, 30, 31	6	873	18	6	1.00	10.52	6	411	9	3	0.73	12.17	12	683	4	5	0.58	1.16	12	423	1	2	0.17	0.52	12	342	2	3	0.67	3.44
			754	8.3	2	1.00	11.01		391	4.94	1.78	0.78	13.07		576	0.63	1.54	0.54	1.09		323	0.21	1.21	0.21	0.65		248	1.45	2.33	0.62	3.25
**10**	29	2	873	2	2	1.00	2.65	3	411	1	2	0.67	1.76	4	683	2	2	0.50	1.74	4	423	1	2	0.50	1.55	6	342	1	2	0.53	2.16
			754	-	-	-	-		391	0.67	1.67	0.67	1.77		576	1	1.5	0.50	1.74		323	0.49	1.49	0.49	1.52		248	0.91	1.91	0.52	2.11
**11**	35, 36	4	873	6	4	1.00	4.42	4	411	1	2	0.50	1.32	8	683	2	3	0.71	1.49	8	423	0	1	0.00	0.00	8	342	1	2	0.43	1.74
			754	3.28	2	1.00	4.35		391	0.52	1.52	0.52	1.38		576	0.85	1.71	0.71	1.48		323	0	1	0.00	0.00		248	0.76	1.76	0.41	1.67
**12**	46	1	873	-	-	-	-	1	411	-	-	-	-	0	-	-	-	-	-	0	-	-	-	-	-	0	-	-	-	-	-
			754						391	-	-	-	-		-	-	-	-	-		-	-	-	-	-		-	-	-	-	-
**13**	40, 41	2	873	3	2	1.00	3.98	2	411	2	2	1.00	5.29	4	683	1	2	0.50	0.87	4	423	2	3	0.83	3.10	4	342	0	1	0.00	0.00
			754	-	-	-	-		391	-	-	-	-		576	0.52	1.52	0.52	0.90		323	1.05	1.88	0.88	3.25		248	-	-	-	-
**14**	42-45	3	873	16	3	1.00	14.15	4	411	10	4	1.00	14.11	4	683	1	2	0.50	0.87	6	423	0	1	0.00	0.00	6	342	0	1	0.00	0.00
			754	10.17	2	1.00	13.49		391	5.26	2	1.00	13.92		576	0.51	1.51	0.51	0.89		323	0	1	0.00	0.00		248	0	1	0.00	0.00
**15**	48-52	9	873	11	9	1.00	4.86	7	411	0	1	0.00	0.00	18	683	0	1	0.00	0.00	18	423	2	3	0.45	2.45	18	342	0	1	0.00	0.00
			754	3.85	2	1.00	5.11		391	0	1	0.00	0.00		576	0	1	0.00	0.00		323	0.8	1.45	0.45	2.48		248	0	1	0.00	0.00
**16**	47	1	873	-	-	-	-	1	411	-	-	-	-	2	683	0	1	0.00	0.00	2	423	0	1	0.00	0.00	0	-	-	-	-	-
			754						391	-	-	-	-		576	-	-	-	-		323	-	-	-	-		-	-	-	-	-
**17**	53	3	873	2	3	1.00	1.77	2	411	0	1	0.00	0.00	6	683	0	1	0.00	0.00	4	423	2	2	0.67	4.13	6	342	0	1	0.00	0.00
			754	1.26	2	1.00	1.67		391	-	-	-	-		576	0	1	0.00	0.00		323	1.34	1.67	0.67	4.15		248	0	1	0.00	0.00
		79	873	142	74	1.00	28.21	81	411	83	42	0.97	40.05	156	688	24	21	0.37	0.92	154	423	11	13	0.57	2.40	154	342	13	12	0.73	6.67
**Region**	**Clades**																														
**Balkans**	12, 13, 14	7	873	53	7	1	27.92	8	411	38	8	1	36.66	10	683	2	3	0.38	0.69	12	423	3	4	0.56	1.97	10	342	0	1	0	0.00
			754	-	-	-	-		391	-	-	-	-		576	-	-	-	-		323	-	-	-	-		248	-	-	-	-
**Italy**	7, 8, 9, 10	14	873	60	14	1	27.49	16	411	43	10	0.94	35.45	28	683	8	8	0.44	0.99	28	423	3	5	0.57	2.19	30	342	6	6	0.62	3.24
			754	47.88	7	1	27.25		391	34.87	6.48	9.42	35.35		576	2.6	3.31	0.41	0.90		323	2.58	3.85	0.56	2.17		248	2.76	3.47	0.62	3.20
**Iberia**	1*, 2, 3	24	873	32	22	0.99	15.29	25	411	21	9	9.78	18.94	50	683	8	6	0.19	0.56	46	423	2	3	0.13	0.40	46	342	3	3	0.58	4.56
			754	24.84	6.89	0.99	14.75		391	17.66	4.7	0.79	19.25		576	1.61	2.06	0.20	0.56		323	0.77	1.77	0.14	0.45		248	2.68	2.69	0.58	4.62

N, sample size (in number of gene copies), *ns* number of sites, *S*, number of segregating sites, *h* number of haplotypes, *Hd* haplotype diversity, π nucleotide diversity. For the diversity measures *S*, *h*, *Hd*, and *π* values calculated under a resampling approach are also reported below values calculated on the original datasets. For the number of sites, for each case the first row represents the size of the alignment while the second row represents the number of sites after discarding Ns or unphased positions; “-“ means that no resampling was conducted for that set. *excluding locality 1.

The phylogenetic relationships inferred from the ML tree based on the combined mitochondrial sequences showed 17 well supported clades (BP≥90) with a geographic coherence (Figure 1A). The Bayesian tree showed an identical topology at supported nodes (not shown) with the same 17 clades supported with a posterior probability of 1.00 (Figure 1A), independently of the choice of tree prior. Clade 1 grouped haplotypes from Northwest France and Southeast Pyrenees (Spain). Haplotypes included in clades 2 and 3 occurred in populations from Northern and Central Spain, respectively. Clade 6 is restricted to Provence, France. Five different clades occurred in Italy: clade 5 in Northern Italy beneath the Alpine arch; clades 9 and 10 in central Italy; and clades 7 and 8 in the Calabrian Peninsula. Slovenian populations clustered in clade 11 and samples from Serbia in clade 4. In the Balkan Peninsula, clades 12, 13, and 14 were distributed in Thessaly, Central Greece, and Peloponnese regions, respectively. Clade 16 is restricted to Samothraki Island and clades 15 and 17 are distributed in Turkish Thrace and North-Western Anatolia, respectively. Clades 12 and 16 were formed by single haplotypes, and as such they cannot be strictly considered as clades. However, we use the term ‘clade’ for simplicity, given their divergence from other clades, and, in the case of clade 12, also because this is indeed formed by multiple haplotypes in the analyses including Genbank sequences (see below and Figure 1B). The average uncorrected genetic distance between mitochondrial clades was 4.5 and 3.1% for *cytb* and *nd4* respectively (ranging from 1 to 7.4% for *cytb* and 0.9 to 4.7% for *nd4*) and both loci show significant *F*_ST_ values (0.86 and 0.83 for *cytb* and *nd4* respectively; *P*=0.001). Clades 15, 16, and 17 showed the lowest pairwise distance values at both *cytb* (1%) and *nd4* (0.9%), while the minimum value of pairwise genetic distances across other clades was 1.8% for both genes. Phylogenetic relationships between Iberian clades West of the Pyrenees (clades 2 and 3), between southern Italian clades (clades 7 and 8), between Slovenian and Balkan clades (clades 11 and 12), and among clades from Turkey and Samothraki (clades 15–17) were well supported in both ML and BA trees, while the relationships between clades 1–6 was supported in BA but not in ML analyses (BP<60). The relationships between the remaining clades were not resolved.

The ML analysis combining the 84 *cytb* sequences generated in this study and 103 sequences published from previous studies showed 23 well supported clades (Figure 1B). These included the 17 clades identified based on the combined *cytb*/*nd4* ML tree and are numbered according to the combined ML tree (Figure 1A) and named according to previous studies [22,26,27]. Previous studies had identified 12 of these mitochondrial mtDNA lineages – eight across Italy (“1Aa”, “1Ac”, “1Ad”, “1Ae”, “1B”, “2a”, “2b”, and “2c”; [26]), one from the island of Elba [27], and one from each of Western France, Eastern France, and the Balkans [23]. We separated the clades “1A” and “1B” listed in [26] into their constituent sub-clades, since clade “1A” was not supported by phylogenetic analyses and clade “1B” from southern Italy is composed of distinct units (from Calabria, Apulia and Campania), which are as differentiated as the other clades. In our assessment, ten previously undetected clades were identified (clades 2,3, 8, and 11–17 in Figure 1A). Additionally, a new lineage was identified in East Macedonia (clade 18) that had not been previously described as a group despite being comprised of sequences retrieved from GenBank.

MtDNA TMRCA estimates within *P*. *muralis* were roughly similar independently of the choice of tree prior in BEAST analyses (Additional file 3). The TMRCA of *P*. *muralis* was estimated at 2.47-2.67 million years ago, thus coinciding with the Plio-Pleistocene boundary. The TMRCA estimates of the various clades bound the period from Early Pleistocene to Late Pleistocene.

SAMOVA analyses showed a lack of genetic structure above the clade level (Additional file 4). No clade grouping explained the genetic variation observed in *P*. *muralis* significantly better than other grouping options, as indicated by the uniform increase of *F*_CT_ values toward higher values of *K*.

**Figure 2:**
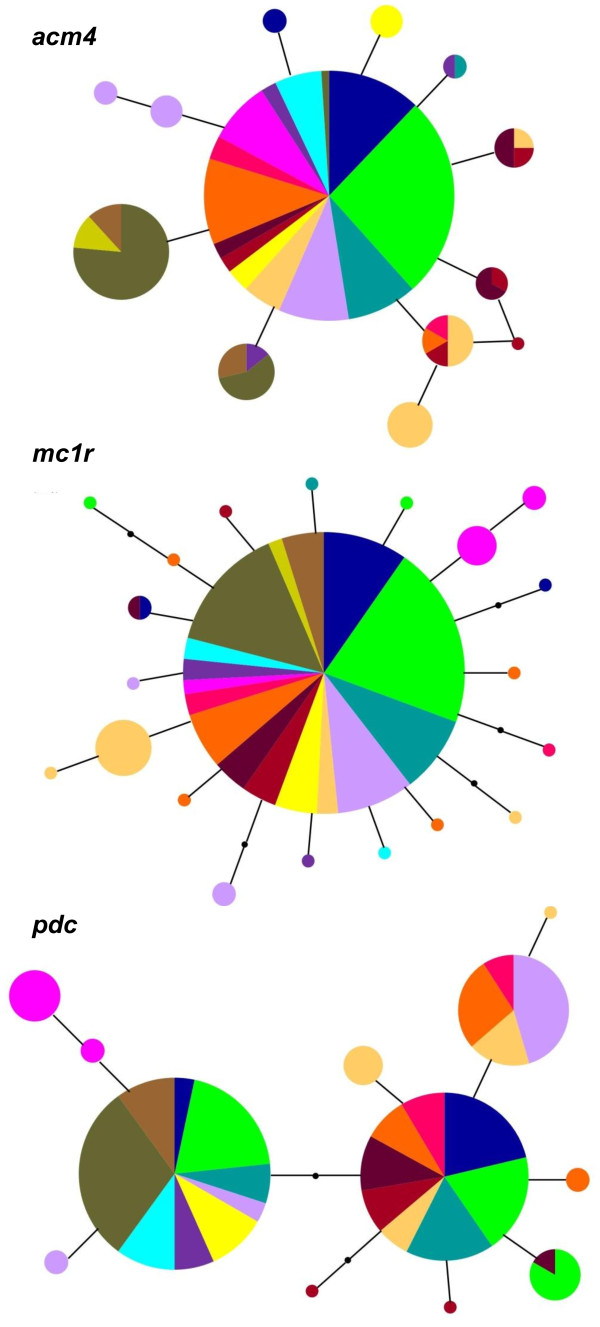
**Phylogenetic relationships among nuclear haplotypes.** Haplotype median joining networks based on sequences of the nuclear genes *acm4*, *mc1r*, and *pdc*. Haplotypes are represented by circles with area proportional to their frequency and coloured according to mitochondrial clades as defined by the Maximum Likelihood tree (see Figure 1).

The phylogenetic networks depicting the relationships between *acm4*, *mc1r*, and *pdc* haplotypes are shown in Figure 2. The *acm4* and *mc1r* networks showed a pattern of low divergence and a lack of apparent phylogeographic structure, although both show significant *F*_ST_ values (0.37 and 0.24 respectively; *P*=0.001) between mtDNA-defined clades. In both loci the most common haplotypes were found in most localities (with the exception of localities 47 and 53 for *acm4*, while locality 46 is not represented in the dataset of these two genes, see Table 1 and 2) and derived haplotypes divergent by one or two mutational steps had a more restricted distribution. On the other hand, evidence of geographic association of nuclear haplotypes was more prominent for the *pdc* locus (Figure 2), leading to a higher and also significant *F*_ST_ value (*F*_ST_ = 0.56; *P*=0.001). The structure of the network showed two most frequent haplotypes, each exhibiting derived variants: the haplogroup placed in the left side of the network was restricted to the Balkans, Turkey, Slovenia, and Provence (France), while the haplogroup on the right side included all the haplotypes from Italy. Haplotypes sampled in the Iberian Peninsula and Eastern France belonged to both haplogroups.

Estimates of genetic diversity *S*, *π*, *H*, and *Hd* for each gene, clade, and geographic region are given in Table 2. Nucleotide and haplotype diversity of mitochondrial genes was higher in Northern, Central, and Southern Italy (clades 5, 9, and 8, respectively) and in clade 14 from Southern mainland Greece and the Peloponnese. At the nuclear gene level, an overall pattern of low genetic diversity was found. Higher diversity values were observed in populations from Northern Italy (clade 5), whereas no clear trend was apparent across putative southern refugial areas.

Permutation tests showed that between-clade *p*-distance is significantly higher than within-clade distances for all loci (*D*_*bc*_/*D*_*wc*_ for mitochondrial DNA: 0.0347/0.0052; for *acm4*: 0.0051/0.0022; for *mc1r*: 0.0029/0.0013; for *pdc*: 0.0097/0.0043; *P*=0.001; see Additional file 5). This allowed us to perform tests aiming at evaluating whether the genetic distance between northern and southern localities is consistent with the distance typically observed either between or within clades. Results from these analyses showed that mean pairwise distances between northern and southern localities are significantly different from within-clade differentiation, but not significantly different from between-clade distance (except locality 1), independently of the locus used for these comparisons (for more details see Additional file 5). Similar results were obtained when comparing northern localities to the sub-set of contiguous southern locations, although in this case this pattern is strong at mitochondrial but less evident at nuclear loci. These results support the hypothesis that populations of *P*. *muralis* from northern regions correspond to distinct evolutionary units from those occurring in southern peninsulas, and therefore that they have likely originated *in situ* rather than as a result of postglacial expansions of southern populations.

## Discussion

The common wall lizard *Podarcis muralis* exhibited a complex phylogeographic pattern with multiple divergent mtDNA clades across its range. In total 23 allo- parapatric clades were identified, most of them with a restricted distribution in the Iberian, Italian, and Balkan peninsulas, as well as in Western and Eastern France, Slovenia and Austria, Central Balkans, and North-Western Anatolia. The detection of previously unidentified lineages was evidently due to the extended sampling scheme of this study as compared to earlier assessments, which were either focussed on a regional scale [27,28], or did not sample all clades endemic to the Iberian, Balkan or Anatolian peninsulas [24]. Although the definition of clades is somewhat arbitrary, the lack of resolution of relationships between monophyletic haplogroups, their high divergence, their geographic coherence, and results from the SAMOVA analysis (Figure 1A,B; Additional file 4) indicate that a grouping into fewer lineages would not be practical and does not describe accurately the mitochondrial DNA diversity exhibited by *P*. *muralis*.

The observed phylogeographic structure of *P*. *muralis* does not match the current subspecific division of this species [22,23], as already reported by [27] and [28] for the 11 subspecies occurring in Italy. Regarding the subspecies found outside Italy, many divergent clades fall into *P*. *m*. *brogniardii* (clades 1–3, 5, and 6) and *P*. *m*. *albanica* (clades 12–14, and 18). Though a taxonomic revision is beyond the scope of this study, our results indicate that the current intraspecific taxonomy of *P*. *muralis* reflects local phenotypic varieties rather than distinct evolutionary units.

While mitochondrial data showed high variability and a clear geographic structure of genetic diversity, nuclear data showed both relatively low genetic variability and shallow divergence among populations. This finding is in agreement with allozyme data [49]; see also [26] from which low levels of polymorphism and genetic differentiation were identified (number of alleles per locus, *A* = 1.07; proportion of polymorphic loci, *P* = 0.07; observed heterozygosity, *H*_o_ = 0.028; genetic distance *D*_NEI_ = 0.036; average values from [49]) among 23 populations of *P*. *muralis* attributable to eight mitochondrial lineages described in our study (clades 1, 2, 3, 5, 9, 14, 22, and 23). Despite low differentiation in nuclear markers, *F*_ST_ values between mitochondrial clades are significant for all three nuclear markers and the mean between-clade pairwise distance is significantly different from that observed within clades, indicating that they are significant evolutionary lineages sensu [46]. Furthermore, even though overall nuclear data showed low genetic variation, a weak geographic association of nuclear haplotypes is apparent in sequence data. At the *acm4* and *mc1r* loci, besides a common widespread haplotype, some closely related derived haplotypes, relatively frequent, were found in the Balkans, Turkey, Slovenia, and Eastern France. This geographic association was more evident at the *pdc* locus, where we observed two main haplotype groups: one including all haplotypes from Italy, and the other comprising haplotypes from the Balkans, Turkey, Slovenia, and Eastern France. Both haplogroups also occurred in Spain and Western France. Such shallow phylogeographic patterns at nuclear loci suggest that the divergence between mitochondrial clades has not been long enough for them to reach reciprocal monophyly at the nuclear level [50-52]. According to mitochondrial DNA TMRCA estimates, divergence among lineages is estimated to have occurred since the Early Pleistocene. Taking into account the slower evolutionary rates of nuclear genes, the genetic pattern observed at the mitochondrial and nuclear level is compatible with a scenario of Pleistocene divergence among lineages. Yet, the use of more variable markers such as microsatellites is needed for a full assessment of the nuclear pattern of variation within this species.

The finding of conspicuous genetic divergence among geographic clusters of populations has provided evidence for the existence of cryptic species within other *Podarcis* taxa including the *P*. *erhardii*[53], *P*. *tiliguerta*[31], and *P*. *hispanica*[54] species complexes. Given the extensive genetic structure observed in *P*. *muralis* and the lack of evidence for syntopy between mitochondrial lineages (except in four populations in Italy, see Figure 1), it might be suggested that some form of barrier to gene exchange has existed between distinct lineages. Nevertheless, we have no evidence that *P*. *muralis* constitutes a species complex. Pairwise uncorrected *p*-distances between clades ranges from 1.0 to 7.4% in *cytb* and from 0.9% to 4.7% in *nd4*, which is a level of divergence typically found within (and not between) recognised species of the genus (e.g. *P*. *vaucheri*, [44]). Further, there is no evidence for high differentiation in nuclear markers which would be indicative of long-term independent evolutionary trajectories. While a low divergence level among genetic lineages is not necessarily a synonym of absence of reproductive isolation, the available evidence suggest that, contrary to other *Podarcis* forms, *P*. *muralis* is a single species and not a species complex.

This does not mean, however, that the genetic landscape of the species is uniform. On the contrary, genetic variation in *P*. *muralis* is structured in many distinct lineages across its range. The phylogeographic pattern observed, with many reciprocally monophyletic allo- parapatric lineages having a Pleistocene divergence, suggests a scenario of long-term isolation in multiple ice-age refugia [1,55]. Therefore, the geographic distribution of these genetic lineages is likely to reflect the refugial structure of this species and indicate putative areas which allowed the long-term maintenance of its genetic diversity. Assessments of many widespread European species have recovered a classic pattern of higher genetic diversity and multiple genetic lineages within southern European peninsulas [2]. On one level the pattern of *P*. *muralis* is not an exception: more than three quarters of all lineages are endemic to the Iberian (2 and 3), the Italian (7–10, 19–23) and the Balkan peninsulas (12–14, 18). The simplest explanation for this phylogeographic pattern is one of a large number of refugia occurring not only across southern peninsulas, as pioneer works in phylogeography have demonstrated at a continental scale (e.g. [1]), but also within each peninsula, conforming to the “refugia within refugia” paradigm [56]. Such compartmentalization is particularly evident in Italy. Multiple, related lineages occur in South Italy, whereas other, unrelated lineages occur in central and northern regions. These latter lineages from central and northern regions were considered by [27] as belonging to a main lineage (clade “1A”) leading to the inference of a main refugial area north of Campania and Apulia. However, though this clade was not supported in any of their phylogenetic analyses (BP ≤ 56, BPP = 0.54), in our analyses its constituent lineages are statistically well supported, unrelated, well differentiated one from the other, and parapatrically distributed. Thus, a scenario of multiple *independent* refugia in Central and Northern Italy appears to fit the data better. A similar reasoning suggests the existence of multiple independent refugia in the southern Balkan Peninsula. On the other hand, the southern Italian Peninsula would have acted as an independent biogeographic compartment with four related lineages arising from distinct refugia within this main refugial area. The same situation is observed in the westernmost and easternmost areas of the species range - in the Iberian Peninsula and in Turkey. Here, two or three endemic related lineages, respectively, occupy geographically different regions that likely acted as distinct refugia within a main refugium. It is noteworthy that in Iberia, where the species currently has a discontinuous distribution, genetic discontinuities do not fully correspond to geographic isolates, suggesting recent fragmentation dynamics.

A scenario of survival in multiple refugia within a southern peninsula has been inferred for many species of the continental Europe fauna and detailed studies on amphibians and reptiles provide the best evidence (see [57] for a recent review). For example, there is evidence for multiple Iberian refugia in *Salamandra salamandra*[58], multiple Italian refugia in *Triturus carnifex*[59] and various Balkan refugia in *Lacerta viridis*[60]. To our knowledge, however, *P*. *muralis* is the first reported case of differentiation into multiple refugia within all Mediterranean peninsulas. In fact, within *P*. *muralis*, diversity and divergence levels are not strikingly different among these regions (Table 2), suggesting that all Mediterranean peninsulas significantly and simultaneously contributed to the long-term persistence of this species throughout the Pleistocene glaciations. Therefore, the case of *P*. *muralis* not only conforms to the scenario of “refugia within refugia” [56], but it also sets a new phylogeographic pattern of “refugia within *all* refugia”.

According to the high number of lineages found in southern European peninsulas, the phylogeographic pattern of *P*. *muralis* fits the classical southern refugia model to some extent. However, while for many species of continental Europe these Mediterranean peninsulas were both glacial refugia and source areas for northward postglacial colonization [2,4], in the case of *P*. *muralis* peninsular lineages are not found outside of these areas. This means that southern peninsulas acted as glacial refugia but not as sources for postglacial expansion. A possible explanation for such an unusual pattern is that northern areas outside Mediterranean peninsulas were already occupied by the species. In fact, various distinct lineages of *P*. *muralis* were found outside southern peninsulas, in the Pyrenees and France (clades 1 and 6), Northern Italy (clade 5), Slovenia and Austria (clade 11) and the Central Balkans (clade 4), reaching the highest latitudes of the species’range. Phylogenetic analyses and permution tests showed that these lineages are well differentiated and evolutionarily independent from southern lineages. The occurrence of two allopatric lineages in Western and Eastern France (clades 1 and 6), which are unrelated and considerably divergent from each other and relative to lineages occurring in Iberia (clade 2 and 3), and on the other side of the Alpine arch in North Italy (clade 5), provides evidence for a long-term persistence of *P*. *muralis* in these areas during Pleistocene glaciations, within separate refugia. Considering that the highest genetic diversity of these lineages is found along the southern portion of their range in SW and SE France respectively (data not shown), we can hypothesize that the refugia where these lineages differentiated were located in proximity of the Mediterranean coast and acted as source areas for post-glacial recolonization of Northern France and Germany. This hypothesis has been tested and validated by a recent study focused on *P*. *muralis* populations from France and Luxembourg [61], and it is further supported by palaeoclimatic and phylogeographic studies which indicate southern France as an area of prolonged ecological stability where climatic oscillations were attenuated [62] and where genetic signatures of refugia have been found among multiple species, including plants and animals (e.g. [63,64]). A similar reasoning applies to the allopatric mitochondrial lineages found in Northern Italy, Northern Adriatic, Eastern Slovenia and Austria, and Central Balkans (clades 5, 21, 11, and 4), which are allopatric and reciprocally monophyletic, thus suggesting long-term isolation in multiple ice-age refugia. The long-term persistence of temperate species in Northern Italy (including the Po Plain and the southern foothills of the Alps) and in the Northern Adriatic has been inferred for many amphibians and reptiles in recent phylogeographic studies based on the occurrence of divergent lineages endemic to these areas (e.g., *Hyla intermedia*, *Pelobates fuscus*, *Pelophylax lessonae*, *Triturus carnifex*, *Podarcis sicula*; see [59,65]). Likewise, in the area spanning from the Eastern Alps to the South-Western Pannonian region, palaeobotanical, paleoclimatic, and genetic data indicate that temperate species such as the tree *Fagus sylvatica* and the butterfly *Erebia medusa* survived in isolated refugia during glacial periods [20,21,66,67]. Finally, a refugial area in the central Balkans and south-western Carpathians has already been suggested for many species showing high genetic diversity and distinct lineages in this area where also broad-leaved trees survived glacial periods (e.g. [16,66-68]).

Altogether these phylogeographic, palaeobotanical and palaeoclimatic studies provide evidence for the long-term persistence of temperate organisms outside the traditional refugia in southern peninsulas, and underline the prominent contribution of these northern refugia to the present-day genetic pools of many temperate species.

## Conclusions

In recent decades, the phylogeography of the Western Palaearctic has been mainly centered around the southern refugia model, which sets a sharp dichotomy between Mediterranean peninsulas and Northern Europe, the former being areas of long-term persistence of temperate species and sources for the postglacial assembly of northern biotas. Our data do not strictly conform to this model: a pattern of northern purity was not detected, whereas several evolutionary units endemic to northern regions were identifed. Overall, the occurrence of many reciprocally monophyletic lineages within *P*. *muralis* suggests a history of long-term persistence in multiple glacial refugia across its distribution, providing a paradigm of ice-age survival of temperate species in Mediterranean and extra-Mediterranean refugia [12]. Contrary to previous hypotheses [25,27,28] this species did not occupy most of its present range only recently, either from the Italian Peninsula or from any other southern peninsular refugium.

In the last two decades mounting evidence for glacial survival in northern refugia through the Pleistocene has been found among various organisms representing different biogeographical groups ([12] for a review). These studies suggest that the assembly of northern European biota was not simply the outcome of long-distance postglacial re-colonization from southern peninsulas [6,7]. Rather, extra-Mediterranean refugia allowed the long-term persistence and differentiation of distinct genetic lineages of many organisms in the Western Palaearctic and acted as source areas for post-glacial colonization of Central and Northern Europe [12]. These biogeographic insights in the Western Palaearctic are paralleled by phylogeographic and palaeobotanical evidence for survival during ice ages of populations of temperate organisms in northern refugia located in the Eastern Palaearctic [69], as well as in the Nearctic region [70-72] providing directions for future research in the Holarctic ecozone concerning the role of northern regions as areas of long-term persistence of temperate biotas.

Understanding the relative contribution of Mediterranean and extra-Mediterranean refugia to the current patterns of genetic diversity may allow us to significantly improve our current knowledge regarding glacial refugia dynamics, the extent and the routes of dispersal between subregions and their consequences in terms of evolutionary and demographic processes [9-12,73]. Moreover, our understanding of northern refugial biodiversity has deep consequences for conservation. The survival of temperate biota in northern refugia across a matrix of unsuitable glacial landscapes challenges the view that organisms must have gone extinct in northern regions during glaciations, and suggests that, in many cases, postglacial colonisation routes – and thus migration capacity - tracking the expansion of suitable habitats have been much smaller than previously thought [9-12,68,74]. Our appraisal of past species resilience, and of their ability to track suitable habitats in response to past climatic shifts, is crucial to better forecast their future performance under global climate change scenarios [10,73,75-77], and thus essential for more informed decisions aimed at long-term conservation of biodiversity.

## Competing interests

The authors declare that they have no competing interests.

## Authors’ contributions

All authors conceived and designed the study, participated in data acquisition, and critically revised and approved the final version of the manuscript; DS, CP analysed the data; DS, CP, DJH drafted the manuscript; CP, DJH, MAC provided funding

## Supplementary Material

Additional file 1 **Details on Sequences from GenBank used in the study.** Accession numbers, localities, and references for the 103 *cytb* sequences obtained from GenBank used in the phylogenetic analyses. Click here for file

Additional file 2 **Primers used for amplification and sequencing.** Primers sequences and reference for each gene are reported. Click here for file

Additional file 3 **Times to the most recent common ancestor estimates.** Times to the most recent common ancestor of supported mitochondrial DNA clades within *P*. *muralis* (in million years) estimated in BEAST. Values in parenthesis refer to the lower and higher limits of the 95% highest posterior density interval. Click here for file

Additional file 4 **SAMOVA (Spatial Analysis of Molecular Variance) design and results.** A: Geographic location of mitochondrial clades estimated as the centroid between member localities (see also Figure 1). B: Localities and sequences included in each mitochondrial clade as defined by previous phylogenetic analyses. C: SAMOVA results showing relative fixation indices *F*_CT_ and *F*_SC_ (P < 0.001) for pre-defined value of *K* from 2 to 10 (after *K*=10 structures are not informative as one population at a time is removed from the groups structure). Click here for file

Additional file 5 **Statistical comparison between the hypotheses of southern vs. *in situ *origin of northern localities.** Click here for file

## References

[B1] Hewitt GM The genetic legacy of the Quaternary ice ages Nature 2000 405 907 913 10.1038/35016000 10879524

[B2] Hewitt GM The structure of biodiversity – insights from molecular phylogeography Front Zool 2004 1 4 10.1186/1742-9994-1-4 15679920 PMC544936

[B3] Widmer A Lexer C Glacial refugia: sanctuaries for allelic richness, but not for gene diversity Trends Ecol Evol 2001 16 267 269 10.1016/S0169-5347(01)02163-2 11369091

[B4] Schmitt T Molecular Biogeography of Europe: pleistocene cycles and postglacial trends Front Zool 2007 4 11 10.1186/1742-9994-4-11 17439649 PMC1868914

[B5] Hewitt GM Some genetic consequences of ice ages and their role in divergence and speciation Biol J Linn Soc Lond 1996 58 247 276

[B6] Hewitt GM Post-glacial re-colonization of European biota Biol J Linn Soc Lond 1999 68 87 112 10.1111/j.1095-8312.1999.tb01160.x

[B7] Taberlet P Fumagalli L Wust-Saucy AG Cosson JF Comparative phylogeography and postglacial colonization routes in Europe Mol Ecol 1998 7 453 464 10.1046/j.1365-294x.1998.00289.x 9628000

[B8] Habel JC Schmitt T Müller P The fourth paradigm pattern of post-glacial range expansion of European terrestrial species: the phylogeography of the Marbled White butterfly (Satyrinae, Lepidoptera) J Biogeogr 2005 32 1489 1497 10.1111/j.1365-2699.2005.01273.x

[B9] Stewart JR Lister AM Cryptic northern refugia and the origins of the modern biota Trends Ecol Evol 2001 16 608 613 10.1016/S0169-5347(01)02338-2

[B10] Provan J Bennett KD Phylogeographic insights into cryptic glacial refugia Trends Ecol Evol 2008 23 564 571 10.1016/j.tree.2008.06.010 18722689

[B11] Stewart JR Lister AM Barnes I Dalén L Refugia revisited: individualistic responses of species in space and time Proc R Soc Lond B Biol Sci 2010 277 661 671 10.1098/rspb.2009.1272 PMC2842738 19864280

[B12] Schmitt T Varga Z Extra-Mediterranean refugia: the rule and not the exception? Front Zool 2012 9 22 10.1186/1742-9994-9-22 22953783 PMC3462695

[B13] Deffontaine V Libois R Kotlík P Sommer R Nieberding C Paradis E Searle JB Michaux JR Beyond the Mediterranean peninsulas: evidence of Central European glacial refugia for a temperate forest mammal species, the bank vole (*Clethrionomys glareolus*) Mol Ecol 2005 14 1727 1739 10.1111/j.1365-294X.2005.02506.x 15836645

[B14] Rowe G Harris DJ Beebee TJC Lusitania revisited: a phylogeographic analysis of the natterjack toad *Bufo calamita* across its entire biogeographical range Mol Phylogenet Evol 2006 39 335 346 10.1016/j.ympev.2005.08.021 16230033

[B15] Joger U Fritz U Guicking D Kalyabina-Hauf S Nagy ZT Wink M Phylogeography of western Palaearctic reptiles – Spatial and temporal speciation patterns Zool Anz 2007 246 293 313 10.1016/j.jcz.2007.09.002

[B16] Kotlík P Deffontaine V Mascheretti S Zima J Michaux JR Searle JB A northern glacial refugium for bank voles (*Clethrionomys glareolus*) Proc Natl Acad Sci USA 2006 103 14860 14864 10.1073/pnas.0603237103 17001012 PMC1595441

[B17] Varga Z Habel JC, Assmann T Extra-Mediterranean refugia, post-glacial vegetation history and area dynamics in Eastern Central Europe Relict Species: Phylogeography and Conservation Biology 2010 Heidelberg: Springer 57 87

[B18] Saarma U Ho SY Pybus OG Kaljuste M Tumanov IL Kojola I Vorobiev AA Markov NI Saveljev AP Valdmann H Lyapunova EA Abramov AV Männil P Korsten M Vulla E Pazetnov SV Pazetnov VS Putchkovskiy SV Rõkov AM Mitogenetic structure of brown bears (*Ursus arctos* L.) in northeastern Europe and a new time frame for the formation of European brown bear lineages Mol Ecol 2007 16 401 13 17217353 10.1111/j.1365-294X.2006.03130.x

[B19] Valdiosera CE Garcia N Anderlung C Dalen L Cregut-Bonnoure E Kahlke RD Stiller M Brandström M Thomas MG Arsuaga J-L Götherström A Barnes I Staying out in the cold: glacial refugia and mitochondrial DNA phylogeography in ancient European brown bears Mol Ecol 2007 16 5140 5148 10.1111/j.1365-294X.2007.03590.x 18031475

[B20] Magri D Vendramin GG Comps B Dupanloup I Geburek T Gömöry D Latałowa M Litt T Paule L Roure JM Tantau I van der Knaap WO Petit RJ De Beaulieu JL A new scenario for the Quaternary history of European beech populations: palaeobotanical evidence and genetic consequences New Phytol 2006 171 199 221 10.1111/j.1469-8137.2006.01740.x 16771995

[B21] Magri D Patterns of post-glacial spread and the extent of glacial refugia of European beech (*Fagus sylvatica*) J Biogeogr 2008 35 450 463 10.1111/j.1365-2699.2007.01803.x

[B22] Gruschwitz M Böhme W Böhme W *Podarcis muralis *(Laurenti, 1768) – Mauereidechse Handbuch der Reptilien und Amphibien Europas, Band 2/II, Echsen (Sauria) III (Lacertidae III: Podarcis) 1986 Wiesbaden: Aula-Verlag 155–208

[B23] Biaggini M Bombi P Capula M Corti C Corti C, Capula M, Luiselli L, Razzetti E, Sindaco R *Podarcis muralis* (Laurenti, 1768) Fauna d’Italia, vol.5, Reptilia 2011 Bologna: Calderini 391 401

[B24] Schulte U Veith M Hochkirch A Rapid genetic assimilation of native wall lizard populations (*Podarcis muralis*) through extensive hybridization with introduced lineages Mol Ecol 2012 17 4313 4326 22765844 10.1111/j.1365-294X.2012.05693.x

[B25] Harris DJ Arnold EN Relationships of Wall Lizards, *Podarcis* (Reptilia: Lacertidae) based on Mitochondrial DNA sequences Copeia 1999 3 749 754

[B26] Capula M High genetic variability in insular populations of the lacertid lizard, *Podarcis muralis* Biochem Syst Ecol 1997 25 411 417 10.1016/S0305-1978(97)00013-6

[B27] Giovannotti M Nisi-Cerioni P Caputo V Mitochondrial DNA sequence analysis reveals multiple Pleistocene glacial refugia for *Podarcis muralis* (Laurenti, 1768) in the Italian Peninsula Ital J Zool 2010 77 277 288 10.1080/11250000903143885

[B28] Bellati A Pellitteri-Rosa D Sacchi R Nistri A Galimberti A Casiraghi M Fasola M Galeotti P Molecular survey of morphological subspecies reveals new mitochondrial lineages in *Podarcis muralis* (Squamata: Lacertidae) from the Tuscan Archipelago (Italy) J Zoolog Syst Evol Res 2011 49 240 250 10.1111/j.1439-0469.2011.00619.x

[B29] Salvi D Harris DJ Bombi P Carretero MA Bologna MA Mitochondrial phylogeography of the Bedriaga’s rock lizard, *Archaeolacerta bedriagae* (Reptilia: Lacertidae) endemic to Corsica and Sardinia Mol Phylogenet Evol 2010 56 690 697 10.1016/j.ympev.2010.03.017 20302956

[B30] Barata M Carranza S Harris DJ Extreme genetic diversity in the lizard *Atlantolacerta andreanskyi* (Werner, 1929): a montane cryptic species complex BMC Evol Biol 2012 12 167 10.1186/1471-2148-12-167 22946997 PMC3492105

[B31] Harris DJ Pinho C Carretero MA Corti C Böhme W Determination of genetic diversity within the insular lizard *Podarcis tiliguerta* using mtDNA sequence data, with a reassessment of the phylogeny of *Podarcis* Amphibia-Reptilia 2005 26 401 407 10.1163/156853805774408676

[B32] Pyron RA Burbrink FT Wiens JJ A phylogeny and revised classification of Squamata, including 4161 species of lizards and snakes BMC Evol Biol 2013 13 93 10.1186/1471-2148-13-93 23627680 PMC3682911

[B33] Sambrook J Fritsch EF Maniatis T Molecular Cloning: A Laboratory Manual 1989 2 New York: Cold Spring Harbor Press

[B34] Harris DJ Reassessment of comparative genetic distance in reptiles from the mitochondrial cytochrome *b* gene Herpetol J 2002 12 85 86

[B35] Stephens M Smith NJ Donnelly P A new statistical method for haplotype reconstruction from population data Am J Hum Genet 2001 68 978 989 10.1086/319501 11254454 PMC1275651

[B36] Bruen T Phillipe H Bryant D A quick and robust statis-tical test to detect the presence of recombination Genetics 2006 172 2665 2681 16489234 10.1534/genetics.105.048975 PMC1456386

[B37] Huson DH Bryant D Application of phylogenetic networks in evolutionary studies Mol Biol Evol 2006 23 254 267 16221896 10.1093/molbev/msj030

[B38] Librado P Rozas J DnaSP v5: A software for comprehensive analysis of DNA polymorphism data Bioinformatics 2009 25 1451 1452 10.1093/bioinformatics/btp187 19346325

[B39] Tamura K Peterson D Peterson N Stecher G Nei M Kumar S MEGA5: molecular evolutionary genetics analysis using maximum likelihood, evolutionary distance, and maximum parsimony methods Mol Biol Evol 2011 28 2731 2739 10.1093/molbev/msr121 21546353 PMC3203626

[B40] Excoffier L Laval G Schneider S Arlequin ver. 3.0: an integrated software package for population genetics data analysis Evol Bioinformatics Online 2005 1 47 50 PMC2658868 19325852

[B41] Jobb G TREEFINDER version of October 2008 2008 Germany: Munich Distributed by the author at http://www.treefinder.de

[B42] Drummond AJ Suchard MA Xie D Rambaut A Bayesian phylogenetics with BEAUti and the BEAST 1.7 Mol Biol Evol 2012 29 1969 1973 10.1093/molbev/mss075 22367748 PMC3408070

[B43] Drummond A Ho S Phillips M Rambaut A Relaxed phylogenetics and dating with confidence PLoS Biol 2006 4 699 10.1371/journal.pbio.0040088 PMC1395354 16683862

[B44] Pinho C Harris DJ Ferrand N Contrasting patterns of population subdivision and historical demography in three western Mediterranean lizard species inferred from mitochondrial DNA variation Mol Ecol 2007 16 1191 205 10.1111/j.1365-294X.2007.03230.x 17391406

[B45] Fouquet A Noonan B Rodrigues MT Pech N Gilles A Gemmell NJ Multiple quaternary refugia in the Eastern Guiana Shield revealed by comparative phylogeography of 12 frog species Syst Biol 2012 61 3 461 489 10.1093/sysbio/syr130 22223446

[B46] Moritz C Defining "Evolutionary Significant Units" for conservation Trends Ecol Evol 1994 9 10 373 375 10.1016/0169-5347(94)90057-4 21236896

[B47] Dupanloup I Schneider S Excoffier L A simulated annealing approach to define the genetic structure of populations Mol Ecol 2002 11 2571 2581 10.1046/j.1365-294X.2002.01650.x 12453240

[B48] Bandelt HJ Forster P Rohl A Median-joining networks for inferring intraspecific phylogenies Mol Biol Evol 1999 16 37 45 10.1093/oxfordjournals.molbev.a026036 10331250

[B49] Capula M Corti C Genetic variability in mainland and insular populations of *Podarcis muralis* (Reptilia: Lacertidae) Bonn Zool Bull 2010 57 189 196

[B50] Moore WS Inferring phylogenies from mtDNA variation: mitochondrial-gen trees versus nuclear-gen trees Evolution 1995 49 718 726 10.2307/2410325 28565131

[B51] Palumbi SR Ciprinao F Hare MP Predicting nuclear gene coalescence from mitochondrial data: the three time-rule Evolution 2001 55 859 868 10.1554/0014-3820(2001)055[0859:PNGCFM]2.0.CO;2 11430646

[B52] Hudson RR Turelli M Stochasticity overrules the ‘three-time rule’: genetic drift, genetic draft and coalescence times for nuclear loci *vs*. mtDNA Evolution 2003 57 182 190 12643581 10.1111/j.0014-3820.2003.tb00229.x

[B53] Poulakakis N Lymberakis P Antoniou A Chalkia D Zouros E Mylonas M Valakos E Molecular phylogeny and biogeography of the wall-lizard *Podarcis erhardii* (Squamata: Lacertidae) Mol Phylogenet Evol 2003 28 38 46 10.1016/S1055-7903(03)00037-X 12801470

[B54] Kaliontzopoulou A Pinho C Harris DJ Carretero MA When cryptic diversity blurs the picture: a cautionary tale from Iberian and North African *Podarcis* wall lizards Biol J Linn Soc Lond 2011 103 779 800 10.1111/j.1095-8312.2011.01703.x

[B55] Avise JC Phylogeography: the history and formation of the species 2000 Harvard: University Press

[B56] Gómez A Lunt DH Weiss S, Ferrand N Refugia within refugia: patterns of phylogeographic concordance in the Iberian Peninsula Phylogeography in southern European refugia: evolutionary perspectives on the origins and conservation of European biodiversity 2007 Dordrecht: Springer Verlag 155 188

[B57] Hewitt GM Zachos FE, Habel JC Mediterranean Peninsulas - the evolution of hotspots Biodiversity hotspots 2011 Heidelberg: Springer 123 147

[B58] García-París M Alcobendas M Buckley D Wake DB Dispersal of viviparity across contact zones in Iberian populations of fire salamanders (*Salamandra*) inferred from discordance of genetic and morphological traits Evolution 2003 57 129 143 12643573 10.1111/j.0014-3820.2003.tb00221.x

[B59] Canestrelli D Salvi D Maura M Bologna MA Nascetti G One Species, three Pleistocene evolutionary histories: phylogeography of the Italian crested newt Triturus carnifex. PLoS ONE 2012 7 7 e41754 10.1371/journal.pone.0041754 PMC3406094 22848590

[B60] Böhme MU Fritz U Kotenko T Džukić G Ljubisavljević K Tzankov N Berendonk TU Phylogeography and cryptic variation within the *Lacerta viridis* complex Zool Scr 2007 36 119 131 10.1111/j.1463-6409.2006.00262.x

[B61] Gassert F Schulte U Husemann M Ulrich W Rödder D Hochkirch A Engel E Meyer J Habel JC From southern refugia to the northern range margin: genetic population structure of the common wall lizard, *Podarcis muralis* J Biogeogr 2013 in press, 10.1111/jbi.12109

[B62] Elenga H Peyron O BonneWlle R Jolly D Cheddadi R Guiot J Andrieu V Bottema S Buchet G De Beaulieu JL Hamilton AC Maley J Marchant R Perez-Obiol R Reille M Riollet G Scott L Straka H Taylor D Van Campo E Vincens A Laarif F Jon-son H Pollen-based biome reconstruction for southern Europe and Africa18,000 yr BP J Biogeogr 2000 27 621 634 10.1046/j.1365-2699.2000.00430.x

[B63] Vogel JC Rumsey FJ Schneller JJ Barrett JA Gibby M Where are the glacial refugia in Europe? Evidence from pteridophytes Biol J Linn Soc Lond 1999 66 23 37

[B64] Ursenbacher S Conelli A Golay P Monney J-C Zuffi MAL Thiery G Durand T Fumagalli L Phylogeography of the asp viper (*Vipera aspis*) inferred from mitochondrial DNA sequence data: evidence for multiple Mediterranean refugial areas Mol Phylogenet Evol 2006 38 546 552 10.1016/j.ympev.2005.08.004 16213755

[B65] Litvinchuk SN Crottini A Federici S De Pous P Donaire D Andreone F Kalezić ML Džukić G Lada GA Borkin LJ Rosanov JM Phylogeographic patterns of genetic diversity in the common spadefoot toad, *Pelobates fuscus* (Anura: Pelobatidae), reveals evolutionary history, postglacial range expansion and secondary contact Org Divers Evol 2013 in press, 10.1007/s13127-013-0127-5

[B66] Willis KJ Van Andel T Trees or no trees? The environments of central and eastern Europe during the last glaciation Quat Sci Rev 2004 23 2369 2387 10.1016/j.quascirev.2004.06.002

[B67] Hammouti N Schmitt T Seitz A Kosuch J Veith M Combining mitochondrial and nuclear evidences: a refined evolutionary history of *Erebia medusa* (Lepidoptera: Nymphalidae: Satyrinae) in Central Europe based on the CO1 gene J Zool Syst Evol Res 2010 48 115 125 10.1111/j.1439-0469.2009.00544.x

[B68] Ursenbacher S Schweiger S Tomovic L Crnobrnja-Isailovic J Fumagalli L Mayer W Molecular phylogeography of the nose-horned viper (*Vipera ammodytes*, Linnaeus (1758)): evidence for high genetic diversity and multiple refugia in the Balkan peninsula Mol Phylogenet Evol 2008 46 1116 1128 10.1016/j.ympev.2007.11.002 18267369

[B69] Korsten M Ho SY Davison J Pähn B Vulla E Roht M Tumanov IL Kojola I Andersone-Lilley Z Ozolins J Pilot M Mertzanis Y Giannakopoulos A Vorobiev AA Markov NI Saveljev AP Lyapunova EA Abramov AV Männil P Valdmann H Pazetnov SV Pazetnov VS Rõkov AM Saarma U Sudden expansion of a single brown bear maternal lineage across northern continental Eurasia after the last ice age: a general demographic model for mammals? Mol Ecol 2009 18 1963 1979 10.1111/j.1365-294X.2009.04163.x 19434812

[B70] Jackson ST Webb RS Anderson KH Overpeck JT Webb T III Williams JW Hansen BCS Vegetation and environment in eastern North America during the last glacial maximum Quat Sci Rev 2000 19 489 508 10.1016/S0277-3791(99)00093-1

[B71] Rowe KC Heske EJ Brown PW Paige KN Surviving the ice: Northern refugia and postglacial colonization Proc Natl Acad Sci USA 2004 101 10355 10359 10.1073/pnas.0401338101 15249673 PMC478575

[B72] Pruett CL Winker K Evidence for cryptic northern refugia among high- and temperate-latitude species in Beringia Clim Change 2008 86 23 27 10.1007/s10584-007-9332-6

[B73] Keppel G Van Niel KP Wardell-Johnson GW Yates CJ Byrne M Byrne M Mucina L Schut AGT Hopper SD Franklin SE Refugia: identifying and understanding safe havens for biodiversity under climate change Global Ecol Biogeogr 2012 21 393 404 10.1111/j.1466-8238.2011.00686.x

[B74] Anderson LL Hu FS Nelson DM Petit RJ Paige KN Ice-age endurance: DNA evidence of a white spruce refugium in Alaska Proc Natl Acad Sci USA 2006 103 12447 12450 10.1073/pnas.0605310103 16894151 PMC1567899

[B75] Lawing AM Polly PD Pleistocene climate, phylogeny, and climate envelope models: an integrative approach to better understand species’ response to climate change PLoS One 2011 16 12 e28554 22164305 10.1371/journal.pone.0028554 PMC3229599

[B76] Willis KJ Bhagwat SA Questions of importance to the conservation of global biological diversity: answers from the past Clim. Past Discuss 2010 6 1139 1162 10.5194/cpd-6-1139-2010

[B77] Willis KJ Bennett KD Froyd C Figueroa-Rangel B How can a knowledge of the past help to conserve the future? Biodiversity conservation strategies and the relevance of long-term ecological studies Phil. Trans. R. Soc. B 2007 362 175 186 10.1098/rstb.2006.1977 17255027 PMC2311423

