# Supplementary material for: Persistence across Pleistocene ice ages in Mediterranean and extra-Mediterranean refugia: phylogeographic insights from the common wall lizard
Source: BMC Evol Biol. 2013 Jul 11;13:147. doi: 10.1186/1471-2148-13-147 (PMC3711914; doi:10.1186/1471-2148-13-147)
Supplement: Additional file 1 — Details on Sequences from GenBank used in the study. Accession numbers, localities, and references for the 103 cytb sequences obtained from GenBank used in the phylogenetic analyses. [file 1471-2148-13-147-S1.pdf]

Table S1. Accession numbers, localities, and references for the 103 *cytb* sequences obtained from GenBank and included in the phylogenetic analyses.

Accession Number	Locality	Author	Reference
JX065616	Germany, Wittlich	Schulte et al.	Mol. Ecol. 21: 4313-4326 (2012)
JQ403294	France, Amboise	Schulte et al.	Amphibia-Reptilia 33: 129-140 (2012)
JQ403292	France, La Rochelle	Schulte et al.	Amphibia-Reptilia 33: 129-140 (2012)
JQ403290	France, Montsegur	Schulte et al.	Amphibia-Reptilia 33: 129-140 (2012)
JQ403293	France, St. Malo	Schulte et al.	Amphibia-Reptilia 33: 129-140 (2012)
JQ403291	France, Lourdes	Schulte et al.	Amphibia-Reptilia 33: 129-140 (2012)
JQ403289	France, Labeaume	Schulte et al.	Amphibia-Reptilia 33: 129-140 (2012)
FR821795	Italy, Elba Island	Bellati et al.	J. Zool. Syst. Evol. Res. 49: 240–250
FR821793	Italy, Elba Island	Bellati et al.	J. Zool. Syst. Evol. Res. 49: 240–250
FR821791	Italy, Gorgona Island	Bellati et al.	J. Zool. Syst. Evol. Res. 49: 240–250
FR821789	Italy, Monte Argentario	Bellati et al.	J. Zool. Syst. Evol. Res. 49: 240–250
FR821787	Italy, Borgo Montello	Bellati et al.	J. Zool. Syst. Evol. Res. 49: 240–250
FR821785	Italy, Calci	Bellati et al.	J. Zool. Syst. Evol. Res. 49: 240–250
FR821783	Italy, Bereguardo	Bellati et al.	J. Zool. Syst. Evol. Res. 49: 240–250
FR821781	Italy, Verona	Bellati et al.	J. Zool. Syst. Evol. Res. 49: 240–250
FR821794	Italy, Elba Island	Bellati et al.	J. Zool. Syst. Evol. Res. 49: 240–250
FR821792	Italy, Portoferraio Rock	Bellati et al.	J. Zool. Syst. Evol. Res. 49: 240–250
FR821790	Italy, Monte Argentario	Bellati et al.	J. Zool. Syst. Evol. Res. 49: 240–250
FR821788	Italy, Borgo Montello	Bellati et al.	J. Zool. Syst. Evol. Res. 49: 240–250
FR821786	Italy, Borgo Montello	Bellati et al.	J. Zool. Syst. Evol. Res. 49: 240–250
FR821784	Italy, Pavia	Bellati et al.	J. Zool. Syst. Evol. Res. 49: 240–250
FR821782	Italy, Bereguardo	Bellati et al.	J. Zool. Syst. Evol. Res. 49: 240–250
HQ605833	France, Lac du Saut de Vesoles	Greenbaum et al.	Zool. J. Linn. Soc. 163: 913-942 (2011)
FJ867394	Italy, Pollino	Giovannotti et al.	Ital. J. Zool. 77: 277-288 (2010)
FJ867392	Italy, Pollino	Giovannotti et al.	Ital. J. Zool. 77: 277-288 (2010)
FJ867390	Italy, Monti Alburni	Giovannotti et al.	Ital. J. Zool. 77: 277-288 (2010)
FJ867388	Italy, Matese	Giovannotti et al.	Ital. J. Zool. 77: 277-288 (2010)
FJ867386	Italy, Gargano	Giovannotti et al.	Ital. J. Zool. 77: 277-288 (2010)
FJ867384	Italy, Gargano	Giovannotti et al.	Ital. J. Zool. 77: 277-288 (2010)
FJ867382	Italy, Gran Sasso	Giovannotti et al.	Ital. J. Zool. 77: 277-288 (2010)
FJ867380	Italy, Visso	Giovannotti et al.	Ital. J. Zool. 77: 277-288 (2010)
FJ867378	Italy, Genga	Giovannotti et al.	Ital. J. Zool. 77: 277-288 (2010)
FJ867376	Italy, Pisa	Giovannotti et al.	Ital. J. Zool. 77: 277-288 (2010)
FJ867374	Italy, Latina	Giovannotti et al.	Ital. J. Zool. 77: 277-288 (2010)
FJ867372	Italy, Cesena	Giovannotti et al.	Ital. J. Zool. 77: 277-288 (2010)
FJ867370	Italy, Parma	Giovannotti et al.	Ital. J. Zool. 77: 277-288 (2010)
FJ867368	Italy, Pavia	Giovannotti et al.	Ital. J. Zool. 77: 277-288 (2010)
FJ867366	Italy, Trieste	Giovannotti et al.	Ital. J. Zool. 77: 277-288 (2010)
FJ867393	Italy, Pollino	Giovannotti et al.	Ital. J. Zool. 77: 277-288 (2010)
FJ867391	Italy, Monti Alburni	Giovannotti et al.	Ital. J. Zool. 77: 277-288 (2010)
FJ867389	Italy, Monti Alburni	Giovannotti et al.	Ital. J. Zool. 77: 277-288 (2010)
FJ867387	Italy, Latina	Giovannotti et al.	Ital. J. Zool. 77: 277-288 (2010)
FJ867385	Italy, Gargano	Giovannotti et al.	Ital. J. Zool. 77: 277-288 (2010)
FJ867383	Italy, L'Aquila	Giovannotti et al.	Ital. J. Zool. 77: 277-288 (2010)
FJ867381	Italy, Caramanico	Giovannotti et al.	Ital. J. Zool. 77: 277-288 (2010)
FJ867379	Italy, Monte San Vicino	Giovannotti et al.	Ital. J. Zool. 77: 277-288 (2010)
FJ867377	Italy, Genga	Giovannotti et al.	Ital. J. Zool. 77: 277-288 (2010)
FJ867375	Italy, Ancona	Giovannotti et al.	Ital. J. Zool. 77: 277-288 (2010)
FJ867373	Italy, Cesena	Giovannotti et al.	Ital. J. Zool. 77: 277-288 (2010)
FJ867371	Italy, Ravenna	Giovannotti et al.	Ital. J. Zool. 77: 277-288 (2010)
FJ867369	Italy, Val Germanasca	Giovannotti et al.	Ital. J. Zool. 77: 277-288 (2010)
FJ867367	Italy, Pavia	Giovannotti et al.	Ital. J. Zool. 77: 277-288 (2010)
FJ867365	Italy, Trento	Giovannotti et al.	Ital. J. Zool. 77: 277-288 (2010)
DQ081149	Spain, Guadarrama, Madrid	Pinho et al.	Mol. Phylogenet. Evol. 38: 266-273 (2006)
DQ081150	Spain, Tanes, Asturias	Pinho et al.	Mol. Phylogenet. Evol. 38: 266-273 (2006)
DQ001031	Greece, Akhaia, Zaruchla	Podnar et al.	J. Mol. Evol. 64: 308-320 (2007)
DQ001029	Germany, Offenburg	Podnar et al.	J. Mol. Evol. 64: 308-320 (2007)
DQ001027	Croatia, island Cres	Podnar et al.	J. Mol. Evol. 64: 308-320 (2007)
DQ001025	Croatia, Nova Gradiska, Strmac	Podnar et al.	J. Mol. Evol. 64: 308-320 (2007)
DQ001023	Italy, Calabria, Aspromonte	Podnar et al.	J. Mol. Evol. 64: 308-320 (2007)
DQ001032	Italy, Friuli-Venzia Giulia, Pradielis	Podnar et al.	J. Mol. Evol. 64: 308-320 (2007)
DQ001030	Croatia, Biokovo mts.	Podnar et al.	J. Mol. Evol. 64: 308-320 (2007)
DQ001028	Italy, Tuscany, Firenze	Podnar et al.	J. Mol. Evol. 64: 308-320 (2007)
DQ001026	Greece, Preveza, Koronisia	Podnar et al.	J. Mol. Evol. 64: 308-320 (2007)
DQ001024	Italy, Calabria, Serra San Bruno	Podnar et al.	J. Mol. Evol. 64: 308-320 (2007)
DQ001022	Italy, Mte. Gargano, Foresta Umbra	Podnar et al.	J. Mol. Evol. 64: 308-320 (2007)
AY896146	-	Poulakakis et al.	Mol. Phylogenet. Evol. 37: 845-857 (2005)
AY896144	-	Poulakakis et al.	Mol. Phylogenet. Evol. 37: 845-857 (2005)
AY896142	-	Poulakakis et al.	Mol. Phylogenet. Evol. 37: 845-857 (2005)
AY896140	-	Poulakakis et al.	Mol. Phylogenet. Evol. 37: 845-857 (2005)
AY896138	Greece, Peloponnisos (Mavrovouni)	Poulakakis et al.	Mol. Phylogenet. Evol. 37: 845-857 (2005)
AY896136	Greece, Xanthi (Leivaditis)	Poulakakis et al.	Mol. Phylogenet. Evol. 37: 845-857 (2005)
AY896134	Greece, Thessalia (Kazarma)	Poulakakis et al.	Mol. Phylogenet. Evol. 37: 845-857 (2005)
AY896132	Greece, Sterea Ellada (Vardousia)	Poulakakis et al.	Mol. Phylogenet. Evol. 37: 845-857 (2005)
AY896130	Greece, Peloponnisos (Taygetos)	Poulakakis et al.	Mol. Phylogenet. Evol. 37: 845-857 (2005)
AY896128	Greece, Sterea Ellada (Gkiona)	Poulakakis et al.	Mol. Phylogenet. Evol. 37: 845-857 (2005)
AY896126	Greece, Makedonia (Ag. Germanos)	Poulakakis et al.	Mol. Phylogenet. Evol. 37: 845-857 (2005)
AY896145	-	Poulakakis et al.	Mol. Phylogenet. Evol. 37: 845-857 (2005)
AY896143	Greece, Thessalia (Sarantaporo)	Poulakakis et al.	Mol. Phylogenet. Evol. 37: 845-857 (2005)
AY896141	Greece, Sterea Ellada (Velouxi)	Poulakakis et al.	Mol. Phylogenet. Evol. 37: 845-857 (2005)
AY896139	Greece, Thessalia (Sxizokaravo)	Poulakakis et al.	Mol. Phylogenet. Evol. 37: 845-857 (2005)
AY896137	Greece, Peloponnisos (Mainalo)	Poulakakis et al.	Mol. Phylogenet. Evol. 37: 845-857 (2005)
AY896135	Greece, Xanthi (Kotili)	Poulakakis et al.	Mol. Phylogenet. Evol. 37: 845-857 (2005)
AY896133	-	Poulakakis et al.	Mol. Phylogenet. Evol. 37: 845-857 (2005)
AY896131	-	Poulakakis et al.	Mol. Phylogenet. Evol. 37: 845-857 (2005)
AY896129	-	Poulakakis et al.	Mol. Phylogenet. Evol. 37: 845-857 (2005)
AY896127	-	Poulakakis et al.	Mol. Phylogenet. Evol. 37: 845-857 (2005)
AY896125	Greece, Peloponnisos (Kilini)	Poulakakis et al.	Mol. Phylogenet. Evol. 37: 845-857 (2005)
AY714980	-	Surget-Groba et al.	Biol. J. Linn. Soc. Lond. 87: 1-11 (2006)
AF080278	France (Near Cannes)	Harris et al.	Proc. R. Soc. Lond., B 265: 1939-1948 (1998)
AY151911	Spain, Navacerrada, Madrid	Carranza et al.	System. Biodivers. 2: 57-77 (2004)
AY151909	Andorra	Carranza et al.	System. Biodivers. 2: 57-77 (2004)
AY151912	Spain, Somiedo, Asturias	Carranza et al.	System. Biodivers. 2: 57-77 (2004)
AY151910	Spain, Navacerrada, Madrid	Carranza et al.	System. Biodivers. 2: 57-77 (2004)
AY151908	Andorra	Carranza et al.	System. Biodivers. 2: 57-77 (2004)
AY234155	Spain, Benasque, Huesca	Busack et al.	Amphibia-Reptilia 26: 239-256 (2005)
AF486233	Greece, Peloponnisos (Kalavrita)	Poulakakis et al.	Mol. Phylogenet. Evol. 28: 38-46 (2003)
AY185096	Austria, Baden	Podnar et al.	Unpublished
AF486232	Greece, Thessalia (Kisavos mt)	Poulakakis et al.	Mol. Phylogenet. Evol. 28: 38-46 (2003)
AF248007	France, Souprosse	Surget-Groba et al.	Mol. Phylogenet. Evol. 18: 449-459 (2001)
AY585686	Greece, Corfu City	Mayer & Hill	Unpublished
DQ001020	Austria, Lower Austria, Gumpolds-kirchen	Podnar et al.	J. Mol. Evol. 64: 308-320 (2007)
FJ460597	Austria, Lower Austria	Podnar et al.	Submitted
