# Supplementary material for: Persistence across Pleistocene ice ages in Mediterranean and extra-Mediterranean refugia: phylogeographic insights from the common wall lizard
Source: BMC Evol Biol. 2013 Jul 11;13:147. doi: 10.1186/1471-2148-13-147 (PMC3711914; doi:10.1186/1471-2148-13-147)
Supplement: Additional file 2 — Primers used for amplification and sequencing. Primers sequences and reference for each gene are reported. [file 1471-2148-13-147-S2.pdf]

Table S2. Primers used for amplification and sequencing.

Gene	Primer	Primer sequence	Reference
nd4	ND4	5’ CAC CTA TGA CTA CCA AAA GCT CAT GTA GAA GC 3’	Arévalo et al. (1994)
	Leu	5’ CAT TAC TTT TAC TTG GAA TTT GCA CCA 3’	
cytb	GluDG	5’ TGA CTT GAA RAA CCA YCG TTG 3’	Palumbi et al. (1991)
	Cytb2	5’ CCC TCA GAA TGA TAT TTG TCC TCA 3’	
mc1r	MC1R-F	5’ GGCNGCCATYGTCAAGAACCGGAACC 3’	Pinho et al. (2010)
	MC1R-R	5’ CTC CGR AAG GCR TAG ATG ATG GGG TCC AC 3’	
pdc	PDCPodF	5' AGT ATC GCA AGC GTT GTA TGC AGG 3'	This study
	PDCPodR	5' CCC AGC AAA AAA CTC CTC ACT GAA 3'	
acm4	Tg-F	5’ CAA GCCTGA GAG CAA RAA GG 3’	Gamble et al. (2008)
	Tg-R	5’ ACY TGA CTC CTG GCA ATG CT 3’	

Reference

Arévalo E, Davis SK, Sites JW Jr, 1994. Mitochondrial DNA sequence divergence and phylogenetic relationships among eight chromosome races of the *Sceloporus grammicus* complex (Phrynosomatidae) in central Mexico. Systematic Biology 43, 387-418.

Gamble T, Bauer AM, Greenbaum E, Jackman TR, 2008. Evidence of Gondwanan vicariance in an ancient clade of geckos. Journal of Biogeography 35, 88-104.

Palumbi S, Martin A, Romano S, McMillan WO, Stice L, Grabowski G, 1991. The Simple Fool‟s Guide to PCR. Department of Zoology and Kewalo Marine Laboratory, University of Hawaii, Honolulu.

Pinho C, Rocha S, Carvalho BM, Lopes S, Mourão S, Vallinoto M, Brunes TO, Haddad CFB, Gonçalves H, Sequeira F, Ferrand N., 2010. New primers for the amplification and sequencing of nuclear loci in a taxonomically wide set of reptiles and amphibians. Conservation Genetics Resources 2, 181-185.
