# Supplementary material for: Persistence across Pleistocene ice ages in Mediterranean and extra-Mediterranean refugia: phylogeographic insights from the common wall lizard
Source: BMC Evol Biol. 2013 Jul 11;13:147. doi: 10.1186/1471-2148-13-147 (PMC3711914; doi:10.1186/1471-2148-13-147)
Supplement: Additional file 3 — Times to the most recent common ancestor estimates. Times to the most recent common ancestor of supported mitochondrial DNA clades within P. muralis (in million years) estimated in BEAST. Values in parenthesis refer to the lower and higher limits of the 95% highest posterior density interval. [file 1471-2148-13-147-S3.pdf]

Table S3. Times to the most recent common ancestor of supported clades within *P. muralis* (in million years) estimated in BEAST. Values in parenthesis refer to the lower and higher limits of the 95% highest posterior density interval.

	Tree prior	
**Clade**	**Yule process**	**Coalescent process***
Clade 1	0.57 (0.26-0.96)	0.51 (0.23-0.82)
Clade 2	0.39 (0.20-0.60)	0.32 (0.16-0.49)
Clade 3	0.35 (0.15-0.58)	0.30 (0.12-0.49)
Clade 4	0.85 (0.46-1.29)	0.83 (0.44-1.24)
Clade 5	0.67 (0.33-1.07)	0.65 (0.33-1.03)
Clade 6	0.18 (0.04-0.34)	0.14 (0.03-0.28)
Clade 7	0.22 (0.07-0.41)	0.18 (0.05-0.33)
Clade 8	0.73 (0.36-1.16)	0.76 (0.40-1.17)
Clade 9	1.00 (0.54-1.55)	0.99 (0.55-1.50)
Clade 10	0.17 (0.02-0.37)	0.14 (0.02-0.30)
Clade 11	0.28 (0.09-0.49)	0.23 (0.07-0.40)
Clade 13	0.30 (0.09-0.54)	0.28 (0.09-0.50)
Clade 14	0.89 (0.48-1.36)	0.92 (0.52-1.38)
Clade 15	0.31 (0.14-0.51)	0.25 (0.12-0.40)
Clade 17	0.14 (0.01-0.33)	0.11 (0.01-0.26)
Clades 2+3	1.05 (0.62-1.52)	1.03 (0.64-1.48)
Clades 7+8	1.22 (0.71-1.79)	1.25 (0.73-1.83)
Clades 11+12	1.23 (0.67-1.84)	1.28 (0.76-1.90)
Clades 15+16+17	0.87 (0.49-1.29)	0.82 (0.48-1.20)
Clades 1+2+3+4+5+6	1.80 (1.19-2.47)	1.84 (1.23-2.52)
P. muralis	2.47 (1.66-3.34)	2.67 (1.86-3.68)

* constant size
