# Supplementary material for: Persistence across Pleistocene ice ages in Mediterranean and extra-Mediterranean refugia: phylogeographic insights from the common wall lizard
Source: BMC Evol Biol. 2013 Jul 11;13:147. doi: 10.1186/1471-2148-13-147 (PMC3711914; doi:10.1186/1471-2148-13-147)

Figure S4. SAMOVA (Spatial Analysis of Molecular Variance) design and results. A: Geographic location of mitochondrial clades estimated as the centroid between member localities (see also Fig.1). B: Localities and sequences included in each mitochondrial clade as defined by previous phylogenetic analyses. C: SAMOVA results showing relative fixation indices F_{CT} and F_{SC} (P < 0.001) for pre-defined value of K from 2 to 10 (after K=10 structures are not informative as one population at a time is removed from the groups structure).

A

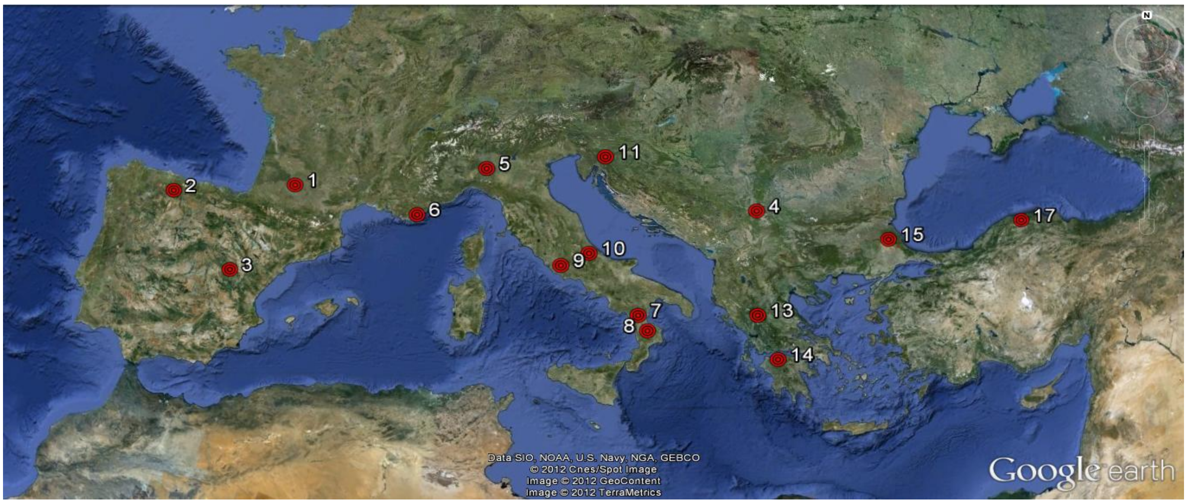

B

Mitochondrial clade	Localities	Samples
**1**	1, 16, 18	DB16840, DB16841, DB14060, DB14063, DB14064, DB11195, DB5030
**2**	2-12	DB4296, DB4296, DB15980, DB15977, DB4281, DB4294, DB4295, DB15968, DB15969, DB15980, DB15970, DB8970, DB8971, DB7092, DB5880, DB5882, DB7065
**3**	13-15	DB8957, DB8958, DB8959, DB13472, DB13473, DB13477, DB13478
**4**	37-39	DB15991, DB15992, DB15993, DB13309, DB16121, DB16122, DB16123
**5**	23, 25, 26	DB16837, DB16838, DPM36, DPM37, DPM38, DPM1, DPM2, DPM3
**6**	20-22	DB13461, DB13460, DB13430
**7**	32, 33	DPM25, DB5937, DPM21
**8**	34	DPM6, DPM7, DPM8
**9**	27, 28, 30, 31	DB1399, DB5938, DPM15, DPM16, DPM18, DPM19
**10**	29	DPM39, DPM40, DPM41
**11**	35, 36	DB13929, DB13930, DB13943, DB13945
**13**	40, 41	DPM31, DPM32
**14**	42-45	DB16934, DPM34, DPM35, DB16936
**15**	48-51	DB16310, DB16307, DB16311, DB16317, DB16482, DB16322, DB16332, DB16485
**17**	53	DB16341, DB16451

C

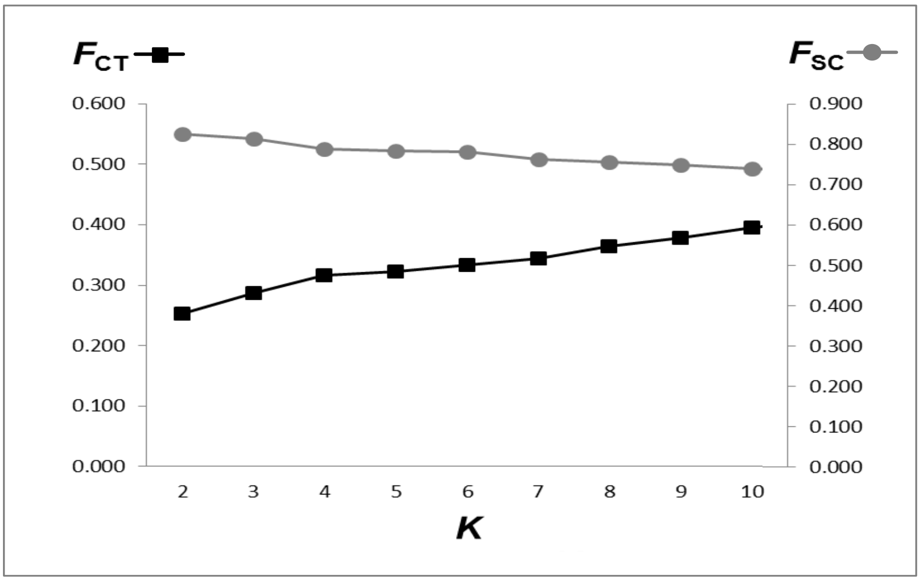

Supplement: Additional file 4 — SAMOVA (Spatial Analysis of Molecular Variance) design and results. A: Geographic location of mitochondrial clades estimated as the centroid between member localities (see also Figure 1). B: Localities and sequences included in each mitochondrial clade as defined by previous phylogenetic analyses. C: SAMOVA results showing relative fixation indices FCT and FSC (P < 0.001) for pre-defined value of K from 2 to 10 (after K=10 structures are not informative as one population at a time is removed from the groups structure). [file 1471-2148-13-147-S4.pdf]
