# Supplementary material for: Persistence across Pleistocene ice ages in Mediterranean and extra-Mediterranean refugia: phylogeographic insights from the common wall lizard
Source: BMC Evol Biol. 2013 Jul 11;13:147. doi: 10.1186/1471-2148-13-147 (PMC3711914; doi:10.1186/1471-2148-13-147)
Supplement: Additional file 5 — Statistical comparison between the hypotheses of southern vs. in situ origin of northern localities. [file 1471-2148-13-147-S5.pdf]

Table S4. Statistical comparison between the hypotheses of a southern vs. in situ origin of northern localities. See text for details.

		**Mitochondrial DNA**			***acm4***			***mc1r***			***pdc***		
	D_{bc}	0.0347			0.0051			0.0029			0.0097		
	D_{wc}	0.0052			0.0022			0.0013			0.0043		
	**Locality**	D_i	$D_i \geq D_{wc}$	$D_i \leq D_{bc}$	D_i	$D_i \geq D_{wc}$	$D_i \leq D_{bc}$	D_i	$D_i \geq D_{wc}$	$D_i \leq D_{bc}$	D_i	$D_i \geq D_{wc}$	$D_i \leq D_{bc}$
Comparison to all southern localities	1	0.0290	***	**	0.0030	ns	**	0.0037	***	ns	0.0069	*	***
	20	0.0359	***	ns	0.0053	ns	ns	0.0030	*	ns	0.0086	**	ns
	21	0.0342	***	ns	0.0064	*	ns	0.0043	**	ns	0.0086	**	ns
	22	0.0360	***	ns	0.0064	*	ns	0.0031	*	ns	0.0086	**	ns
	23	0.0328	***	ns	0.0081	***	ns	0.0034	**	ns	0.0083	***	ns
	25	0.0310	***	ns	0.0075	***	ns	0.0037	***	ns	0.0104	***	ns
	26	0.0314	***	ns	0.0069	***	ns	0.0040	***	ns	0.0109	***	ns
	35	0.0349	***	ns	0.0030	ns	**	0.0035	***	ns	0.0145	***	ns
	36	0.0351	***	ns	0.0030	ns	**	0.0037	***	ns	0.0145	***	ns
	37	0.0316	***	ns	0.0049	**	ns	0.0034	***	ns	0.0096	***	ns
	38	0.0319	***	ns	0.0030	ns	*	0.0030	*	ns	0.0086	**	ns
	39	0.0310	***	*	0.0062	***	ns	0.0043	***	ns	0.0088	***	ns
Comparison to contiguous southern localities	1	0.0230	***	***	0.0025	ns	***	0.0045	***	ns	0.0050	ns	***
	20	0.0411	***	ns	0.0071	**	ns	0.0021	ns	ns	0.0149	***	ns
	21	0.0395	***	ns	0.0081	**	ns	0.0036	*	ns	0.0149	***	ns
	22	0.0414	***	ns	0.0081	**	ns	0.0022	ns	ns	0.0149	***	ns
	23	0.0364	***	ns	0.0082	***	ns	0.0025	*	ns	0.0057	ns	***
	25	0.0371	***	ns	0.0074	***	ns	0.0026	*	ns	0.0071	*	*
	26	0.0373	***	ns	0.0060	***	ns	0.0030	**	ns	0.0080	***	*
	35 [1]	0.0305/0.0347	***/***	ns/ns	0.0005/0.0048	ns/*	***/ns	0.0040/0.0028	**/*	ns/ns	0.0059/0.0210	ns/***	**/ns
	36 [1]	0.0314/0.0354	***/***	ns/ns	0.0005/0.0048	ns/*	***/ns	0.0033/0.0027	**/*	ns/ns	0.0059/0.0210	ns/***	**/ns
	37	0.0372	***	ns	0.0028	ns	*	0.0008	ns	***	0.0108	***	ns
	38	0.0386	***	ns	0.0005	ns	***	0.0004	ns	***	0.0147	***	ns
	39	0.0370	***	ns	0.0045	*	ns	0.0021	ns	*	0.0103	***	ns

Legend: D_{bc} : mean between-clade pairwise distance; D_{wc} : mean within-clade pairwise distance; D_i : mean pairwise distance to southern localities;
* p<0.05; ** p<0.01 ***p<0.001; [1] the double results for these localities correspond to the comparison to Balkan/Italian localities, respectively.
